# ADAMTSL3 knock-out mice develop cardiac dysfunction and dilatation with increased TGFβ signalling after pressure overload

**DOI:** 10.1038/s42003-022-04361-1

**Published:** 2022-12-20

**Authors:** Karoline B. Rypdal, A. Olav Melleby, Emma L. Robinson, Jia Li, Sheryl Palmero, Deborah E. Seifert, Daniel Martin, Catelyn Clark, Begoña López, Kristine Andreassen, Christen P. Dahl, Ivar Sjaastad, Theis Tønnessen, Mathis K. Stokke, William E. Louch, Arantxa González, Stephane Heymans, Geir Christensen, Suneel S. Apte, Ida G. Lunde

**Affiliations:** 1 Institute for Experimental Medical Research, Oslo University Hospital and University of Oslo, Oslo, Norway; 2 Division of Diagnostics and Technology, Akershus University Hospital, Lørenskog, Norway; 3 K.G. Jebsen Center for Cardiac Biomarkers, University of Oslo, Oslo, Norway; 4 Department of Molecular Medicine, Institute of Basic Medical Sciences, University of Oslo, Oslo, Norway; 5 Department of Cardiology, Maastricht University, CARIM School for Cardiovascular Diseases, Maastricht, Netherlands; 6 Department of Biomedical Engineering, Cleveland Clinic Lerner Research Institute, Cleveland, OH USA; 7 Program of Cardiovascular Diseases, CIMA Universidad de Navarra and IdiSNA, Pamplona, Spain; 8 CIBERCV, Carlos III Institute of Health, Madrid, Spain; 9 Department of Cardiology, Oslo University Hospital Rikshospitalet, Oslo, Norway; 10 Department of Cardiothoracic Surgery, Oslo University Hospital Ullevål, Oslo, Norway; 11 Centre for Molecular and Vascular Biology, Department of Cardiovascular Sciences, Leuven, Belgium

**Keywords:** Heart failure, Experimental models of disease, Preclinical research

## Abstract

Heart failure is a major cause of morbidity and mortality worldwide, and can result from pressure overload, where cardiac remodelling is characterized by cardiomyocyte hypertrophy and death, fibrosis, and inflammation. In failing hearts, transforming growth factor (TGF)β drives cardiac fibroblast (CFB) to myofibroblast differentiation causing excessive extracellular matrix production and cardiac remodelling. New strategies to target pathological TGFβ signalling in heart failure are needed. Here we show that the secreted glycoprotein ADAMTSL3 regulates TGFβ in the heart. We found that *Adamtsl3* knock-out mice develop exacerbated cardiac dysfunction and dilatation with increased mortality, and hearts show increased TGFβ activity and CFB activation after pressure overload by aortic banding. Further, ADAMTSL3 overexpression in cultured CFBs inhibits TGFβ signalling, myofibroblast differentiation and collagen synthesis, suggesting a cardioprotective role for ADAMTSL3 by regulating TGFβ activity and CFB phenotype. These results warrant future investigation of the potential beneficial effects of ADAMTSL3 in heart failure.

## Abstract (web summary)

The role of ADAMTSL3 in heart pathology and cardiac disease is investigated with Adamtsl3-deficient mice using CRISPR-Cas9 gene editing.

## Introduction

Heart failure is a major cause of morbidity, hospitalization and mortality worldwide, affecting 2-3% of the population^1^. Upon pressure overload, the myocardium remodels with hallmark pathophysiological responses including cardiomyocyte hypertrophy and death, fibrosis, and inflammation, with hypertrophic growth often preceding dilated heart failure^2^.

The extracellular matrix (ECM) is crucial for maintaining cardiac structural and functional integrity, and cardiac fibroblasts (CFBs) are the main producers of ECM in the heart^3^. During heart failure, CFBs are activated and transdifferentiate into highly proliferating and ECM-producing myofibroblasts^3,4^, characterized by robust expression of α-smooth muscle actin (α-SMA)^5,6^. This process is critical in wound healing, however, excessive CFB activation drives pathological cardiac remodelling through growth factor activation, inflammation, reduced electrical conductance and increased myocardial stiffness, resulting from increased collagen deposition and crosslinking in cardiac fibrosis^3,7^. Transforming growth factor (TGF)β is required for CFB activation^5^ and represents an attractive therapeutic target in heart failure^8^. However, direct TGFβ inhibition is limited by off-target effects^9,10^, and a better understanding of TGFβ-mediated CFB activation in the heart is needed to improve heart failure outcomes.

Matricellular proteins are dynamically expressed, non-structural, regulatory molecules in the cardiac ECM^7^. We recently identified that a seven-membered family of matricellular proteins, the a disintegrin-like and metalloprotease domain with thrombospondin type 1 motifs-like (ADAMTSL) proteins, is upregulated in experimental and clinical heart failure^11^. ADAMTSLs are structurally related to the ADAMTS ECM proteases^12^, but lack a catalytic domain, leaving their biological function largely unknown. Accumulating evidence points to a role in TGFβ regulation, as several ADAMTSLs bind and regulate fibrillin microfibrils and latent TGFβ binding protein (LTBP)1, which sequester TGFβ in the ECM^13,14^. Variants in *ADAMTSL* genes phenocopy fibrillinopathies, with dysregulated TGFβ signalling as a consistent molecular feature^11,15–17^.

Here, we report the generation and characterization of a mouse with targeted inactivation of *Adamtsl3* (L3-KO), which we used to investigate ADAMTSL3 in the pressure-overloaded heart. We found that L3-KO mice develop cardiac dysfunction and dilatation, with higher mortality, and increased TGFβ activity and CFB activation after aortic banding (AB)-induced pressure overload. ADAMTSL3 overexpression in cultured human CFBs inhibited TGFβ activity and myofibroblast conversion, resulting in reduced ECM expression and collagen synthesis.

## Results

### ADAMTSL3 expression is increased in hearts of patients with heart failure and mice with left ventricular pressure overload

**Fig. 1:**
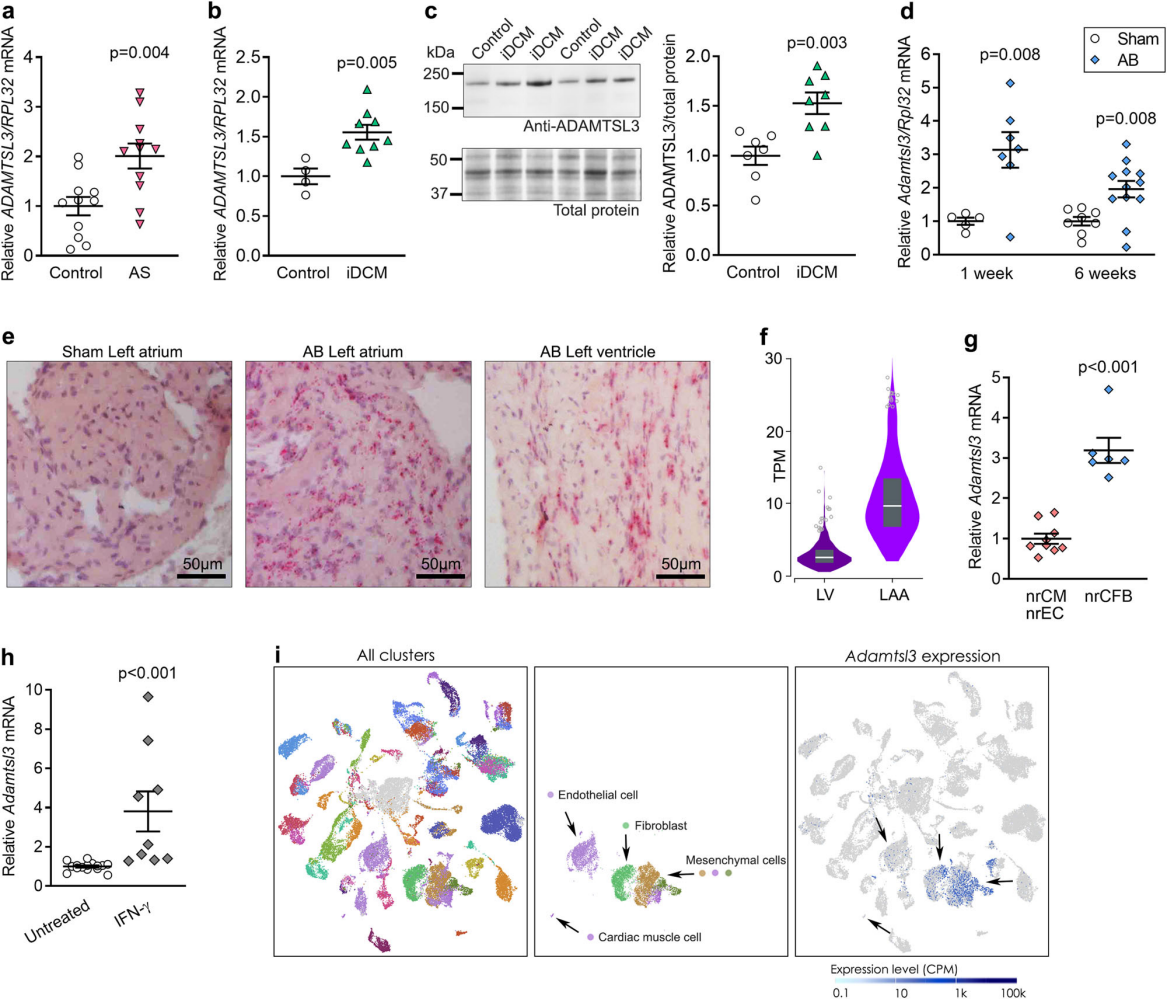
ADAMTSL3 is upregulated in patients with heart failure and in wild-type mice with left ventricular pressure overload, and is produced by cardiac fibroblasts. **a-b** *ADAMTSL3/RPL32* mRNA levels in left ventricular (LV) biopsies from patients with **a** aortic stenosis (AS, *n* = 11) and **b** ischemic dilated cardiomyopathy (iDCM, *n* = 9) vs. respective controls. **c** Western blot and quantification of ADAMTSL3 protein/total protein levels in LVs of patients with iDCM (*n* = 8) vs. controls (*n* = 7). **d** *Adamtsl3/Rpl32* mRNA levels in LVs of wild-type (WT) mice 1 and 6 weeks after aortic banding (AB) or sham surgery (*n* = 5 sham and *n* = 7 AB at 1 week, *n* = 8 sham and *n* = 12 AB at 6 weeks). **e** In situ hybridization of *Adamtsl3* mRNA (red staining) in the left atrium of AB and sham-operated mice, and in the LV wall of AB mice, 4 weeks post-AB, with hematoxylin nuclear counterstain (purple). **f** RNA sequencing data from the Genotype-Tissue Expression (GTEx) project portal. In GTEx, a total of 17382 samples across 54 non-diseased tissues, from 948 deceased human organ donors, represents a control population. Donors are 20-70 years old, 33% female, 85% White and 13% African-American. *ADAMTSL3* expression is shown as transcripts per kilobase million (TPM), in LV (*n* = 432, median TPM = 2.54) and left atrial appendage (LAA, *n* = 429, median TPM = 9.66). **g** mRNA levels of *Adamtsl3* in neonatal rat cardiac fibroblasts (nrCFB) and cardiomyocytes (nrCM), with presence of endothelial cells (nrEC), isolated from 1-3 day old rat hearts (*n* = 3 isolations of *n* = 60 hearts, with *n* = 3 culture replicates per isolation). **h** mRNA levels of *Adamtsl3* in untreated primary nrCFBs, and nrCFBs stimulated with 5 ng IFN-γ. Data are scatterplots (**a**–**d**, **g**–**h**) or violin plot **f** with mean ± SEM. Statistical analyses was performed using the Student’s *t*-test vs. respective controls (**a**–**d**, **g**–**h**). **i** Data from the EMBL-EBI Single Cell Expression Atlas. All clusters with 82 cell types from 20 mouse tissues (*n* = 3 female and *n* = 4 male 10-15 week old mice)^22^, and extraction of endothelial cells, cardiac muscle cells, fibroblasts and mesenchymal cells (fibroblast progenitors) are shown, and the clustering of *Adamtsl3* expression (blue dots). Scale bar is counts per million (CPM) reads mapped.

*ADAMTSL3* expression was upregulated 2-fold in LVs of patients with aortic stenosis (AS; Fig. 1a), and in the myocardium of patients with ischemic DCM (iDCM), *ADAMTSL3* mRNA and protein were upregulated 1.5-fold (Fig. 1b-c). Patient characteristics were previously reported, with patients having symptomatic AS with LV hypertrophy, fibrosis and heart failure with preserved ejection fraction (HFpEF)^18^, or end-stage DCM with fibrosis and dilated heart failure with reduced ejection fraction (HFrEF)^19^. Similarly, *Adamtsl3* expression was increased 2-3-fold in LVs of WT mice subjected to a recently described AB procedure, for one or six weeks, using fixed diameter O-rings^20^ (Fig. 1d). ADAMTSL3 protein levels were not investigated due to lack of working antibodies in mouse (Fig. S1a-b).

### ADAMTSL3 is produced by cardiac fibroblasts

RNA in situ hybridization in WT mouse hearts confirmed *Adamtsl3* expression post-AB in both the atrial and ventricular tissue (Fig. 1e), and showed *Adamtsl3* expression in areas between cardiomyocytes (CMs). Similarly, *ADAMTSL3* was expressed in the LV and left atrial appendage (LAA) (Fig. 1f) of 948 human donors in the Genotype-Tissue Expression (GTEx) database^21^. We obtained primary cultures from neonatal rat hearts where CFBs were separated from CMs, and vascular endothelial cells (ECs) were found in the CM fraction^11^. qPCR analysis revealed high *Adamtsl3* expression in CFBs vs. CMs/ECs (Fig. 1g), and its expression in CFBs was upregulated by interferon-γ (IFN-γ) (Fig. 1h), suggesting that IFN-γ signalling stimulates ADAMTSL3 production in CFBs. Of note, C_T_ values for *Adamtsl3* in the CM/EC fraction were relatively high (Fig. S2), and thus, *Adamtsl3* expression was considered to be very low in the CM/EC fraction. We also mined the Single Cell Expression Atlas database containing published expression data for 82 cell types from 20 mouse tissues^22^. Consistent with data from our primary heart cultures, high *Adamtsl3* mRNA levels were seen in fibroblast and mesenchymal cell (fibroblast progenitors) clusters (Fig. 1i). There was some expression in ECs, while expression was negligible in CMs. Based on this, the *Adamtsl3* signal in the nrCM/nrEC fraction likely originated from ECs. These independent datasets identify fibroblasts as main producers of ADAMTSL3 in human, mouse and rat hearts.

### *Adamtsl3* deletion results in cardiac dysfunction and dilatation in response to pressure overload

**Fig. 2:**
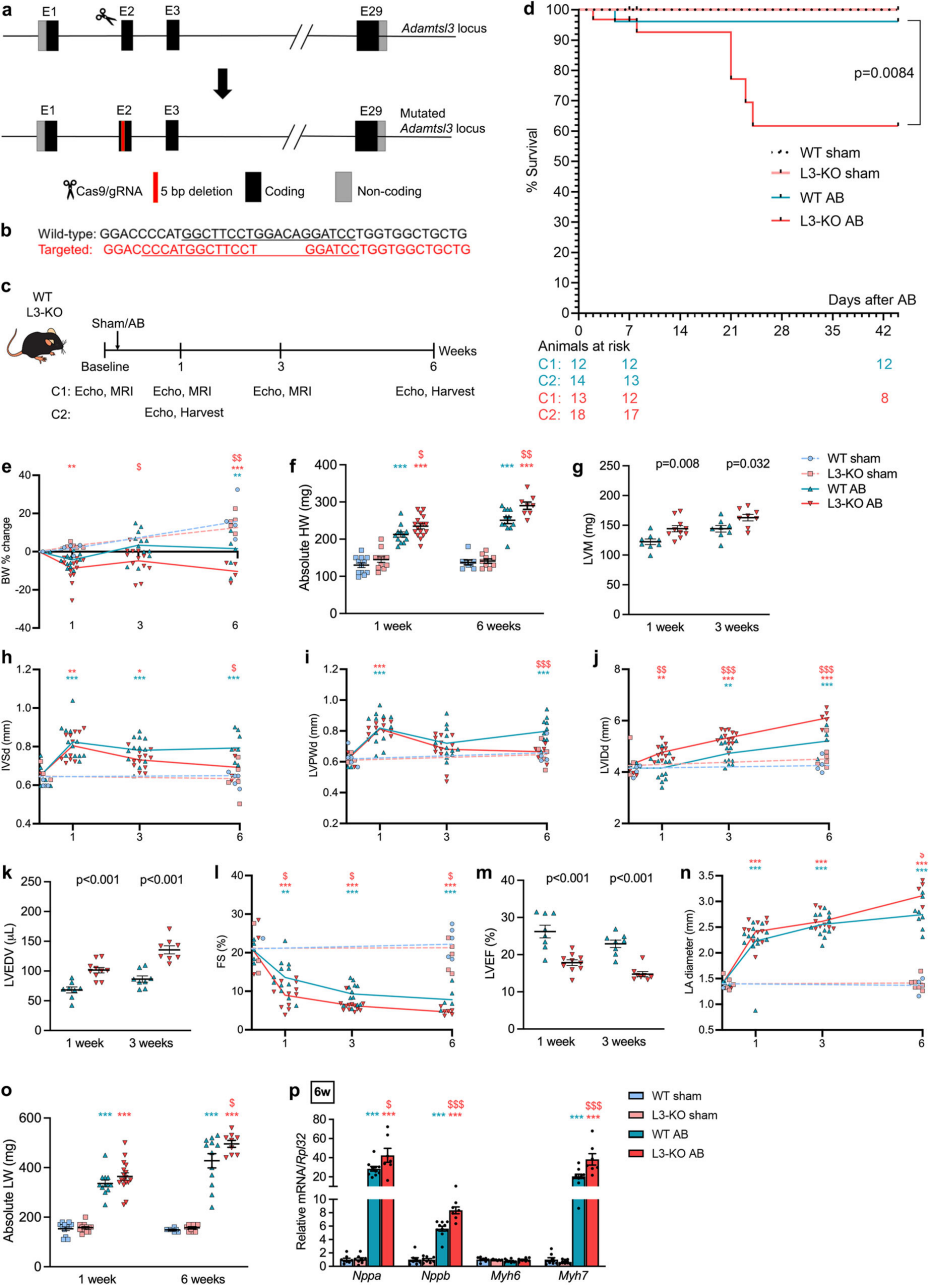
Adamtsl3 inactivation results in contractile dysfunction and cardiac dilatation with high mortality in response to pressure overload. **a**–**b** Generation of the *Adamtsl3* mutant allele. **a** Overview of the *Adamtsl3* locus showing coding and non-coding exons. CRISPR-Cas9 mediated editing resulted in a 5 bp deletion in exon 2. **b** Sequence around the 5 bp deletion target site. The underlined sequences indicate the primers used for genotyping. **c** Schematic timeline of mouse study. Wild-type (WT) and *Adamtsl3* knock-out (L3-KO) mice underwent aortic banding (AB) or sham surgery, and were followed with echocardiography and magnetic resonance imaging (MRI) for 6 weeks. **d** Kaplan-Meier survival curves post-AB. **e** Body weight (BW) change from baseline (%), in WT and L3-KO post-AB or sham surgery (*n* = 8 WT and *n* = 12 L3-KO). **f** Absolute heart weight (HW) post-AB. **g** Left ventricle mass (LVM) post-AB. **h** Interventricular septal thickness in diastole (IVSd), **i** LV posterior wall thickness in diastole (LVPWd), **j** LV inner diameter in diastole (LVIDd), at baseline, and post-AB compared to sham. **k** LV end-diastolic volumes (LVEDV) post-AB. **l** Fractional shortening (FS) at baseline and post-AB. **m** LV ejection fraction (LVEF) post-AB. **n** Left atrial (LA) diameter at baseline compared to sham. **o** Absolute lung weight (LW) in L3-KOs and WTs post-AB. **p** LV atrial and brain natriuretic peptides (*Nppa, Nppb*) and α- and β-myosin heavy chains (*Myh6, Myh7*) 6 weeks post-AB or sham surgery. **e**–**p** *n* = 6 WT and *n* = 6 L3-KO at baseline, *n* = 12 WT and *n* = 8-12 L3-KO post-AB, and n = 7-8 WT and *n* = 9-12 L3-KO post sham surgery. Data are mean ± SEM. Statistical analyses were performed using the Log-rank (Mantel-Cox) test (d), the one-way ANOVA with Tukey’s multiple comparisons test (**e**, **h**–**j**, **l**, **n**, **p**), or the Student’s *t*-test (**f**–**g**, **k**, **m**, **o**). P-values are reported as numeric p-values or *p < 0.05, **p < 0.01, and ***p < 0.001 for AB vs. sham, and ^$^*p* < 0.05, ^$$^*p* < 0.01, and ^$$$^*p* < 0.001 for L3-KO AB vs. WT AB.

Using CRISPR-Cas9-mediated gene editing, an *Adamtsl3* mutant mouse allele was generated (Fig. 2a). Specifically, a 5-bp deletion in exon 2 (Fig. 2b), encoding the signal peptide, resulted in an out-of-frame transcript that eliminated *Adamtsl3* expression. Homozygous and heterozygous mice were distinguishable by *Adamtsl3* genotyping, RNA-seq and qPCR (Fig. S3a-c, respectively). Adult L3-KO mice had an outwardly normal appearance (Fig. S3d), and similar body weight to WT controls (Tables SI-SII). Homozygous L3-KO mice survived into maturity without apparent early lethality, and had apparently normal cardiac dimensions and function, measured by echocardiography and cardiac MRI at 8-12 weeks of age (Table SI). Of note, L3-KO hearts weighed more than WT (9-13%) and heterozygote (13.5%) littermate hearts (Tables SI-SII). L3-KO tibia bones were longer (2-3%) than those of WT littermates, and thus, body weight, which was similar in all three genotypes, was preferred over tibia length for organ weight normalization at baseline (Tables SI-SII).

L3-KO and WT littermates were subjected to increased LV afterload by AB using O-rings of a fixed inner diameter of 0.55 mm, with sham operation performed in corresponding control cohorts^20^. Mice were monitored with echocardiography and MRI at baseline and at one, three and six weeks after operation, and the hearts were harvested at one and six weeks post-AB (Fig. 2c). L3-KO mice had high mortality post-AB, with 62% survival at six weeks, compared to 96% survival of WTs, and 100% survival of sham-operated mice of both genotypes (Fig. 2d). The majority of deceased L3-KO mice died with severe cardiac dysfunction three weeks post-AB. Both genotypes had similar body weight at baseline and lost weight post-AB vs. sham-operated mice, but WTs re-gained more weight than L3-KOs over the post-AB time course (Fig. 2e). At six weeks, the surviving L3-KOs had lost 10% of baseline body weight (Fig. 2e), and thus, the experiment was terminated. This body weight loss was consistent with a more deleterious impact in L3-KO vs. WT post-AB.

Given that L3-KO mice lost more weight than WT mice after AB, organ weights after harvest were not normalized to body weight. Harvested L3-KO hearts weighed more after AB than WT hearts (Fig. 2f), and had greater absolute LV mass as calculated from MRI-measured dimensions (Fig. 2g). Interventricular septal thickness (IVSd) (Fig. 2h) and LV posterior wall thickness (LVPWd) (Fig. 2i), measured by echocardiography, were similar at baseline and increased in both genotypes one-week post-AB, suggesting similar degree of hypertrophic response. At six weeks post-AB, however, while hypertrophy was still evident in WTs, ventricular walls were thinner in L3-KOs and no different from baseline (Fig. 2h-i). LV inner diameter (LVIDd) (Fig. 2j) measured by echocardiography, and LV volumes (LVEDV) (Fig. 2k) measured by MRI, were increased in L3-KOs vs. WTs throughout the study course from one to six weeks post-AB, indicating increased cardiac dilatation of L3-KO compared to WTs (representative echocardiography images are shown in Fig. S4a-b). L3-KOs had reduced fractional shortening (FS) vs. WTs (Fig. 2l) and reduced LVEF (Fig. 2m) from one-week post-AB, indicating reduced contractile function. Left atrial (LA) diameter (Fig. 2n) and lung weights (Fig. 2o) were increased in L3-KO six weeks post-AB, suggesting circulatory congestion. Finally, atrial natriuretic peptide (ANP, *Nppa*), brain natriuretic peptide (BNP, *Nppb*), and β-myosin heavy chain (*Myh7*) expression was increased in L3-KO vs. WT LVs at six weeks (Fig. 2p), consistent with ongoing cardiomyocyte remodelling and dysfunction^23^. Altogether, these data demonstrate that L3-KOs developed exacerbated cardiac dilatation and contractile dysfunction with faster progression to decompensation vs. WTs, upon equally imposed LV afterload.

### RNA sequencing reveals differentially regulated genes in cardiomyopathy, TGFβ, and cardiomyocyte dysfunction pathways in *Adamtsl3* KO hearts after pressure overload

**Fig. 3:**
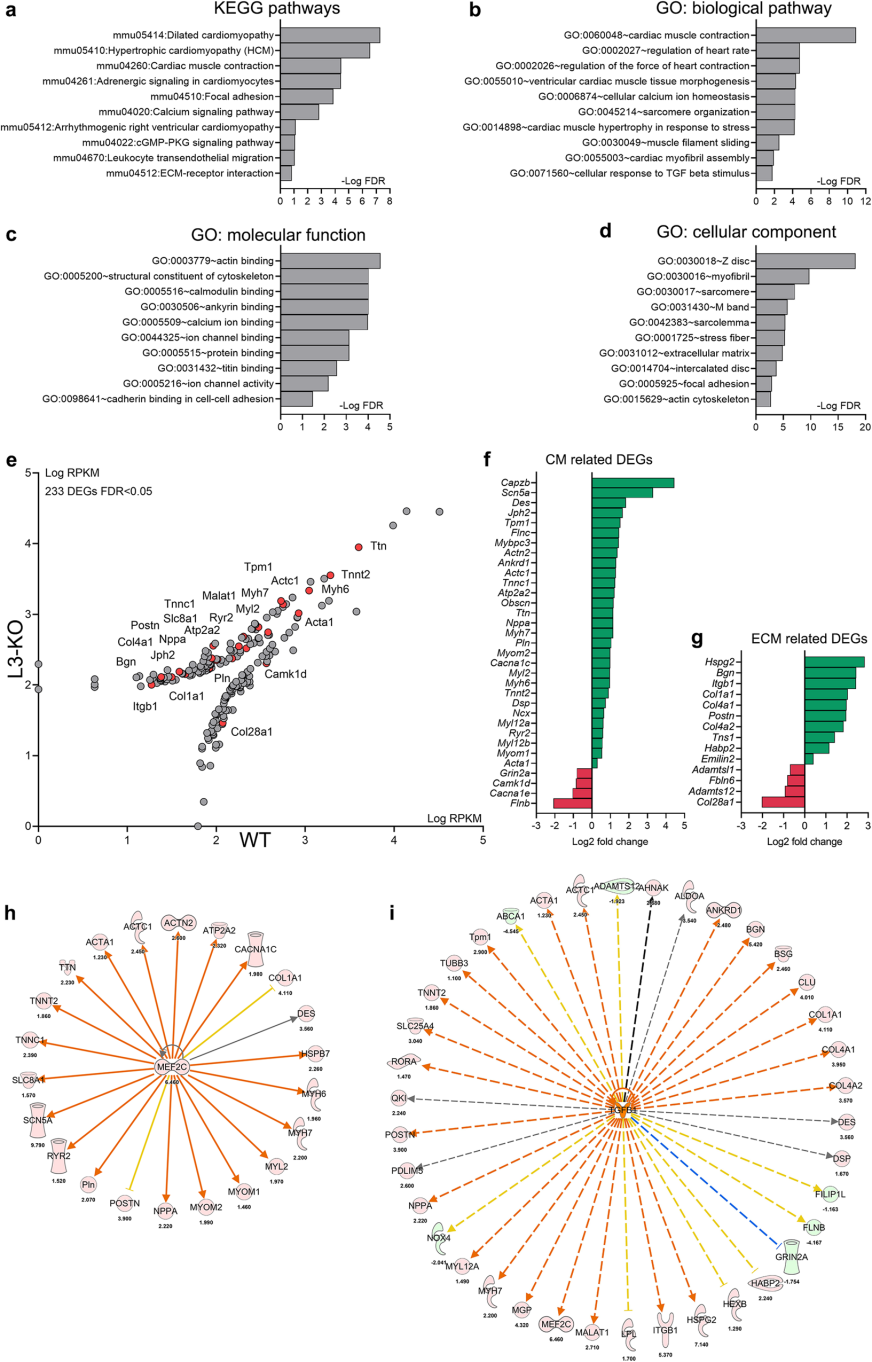
RNA sequencing highlights cardiomyopathy, TGFβ, and cardiomyocyte dysfunction pathways in Adamtsl3 knock-out hearts in response to pressure overload. Pressure overload of the left ventricle (LV) was induced in *Adamtsl3* knock-out (L3-KO, *n* = 10) and wild-type (WT, *n* *=* 6) mice by aortic banding (AB) for 1 week, and RNA sequencing was performed on LV tissue. 233 differentially expressed genes (DEGs, FDR < 0.05) were analysed for enrichment of Kyoto Encyclopedia of Genes and Genomes (KEGG) pathways and Gene Ontology (GO) terms using Database for Annotation, Visualization and Integrated Discovery (DAVID) v6.8 analysis wizard^49^. The 10 most enriched **a** KEGG pathways, **b** GO biological pathways, **c** GO molecular functions, and **d** GO cellular components, identified among the 233 DEGs are shown. **e** Scatterplot comparing log reads per kilobase million (RPKM) of 233 DEGs between L3-KO and WT. Red dots correspond to annotated DEGs in the graph. The complete list of DEGs (gray and red dots) can be found in Supplementary Data 1. **f-g** Log2-fold change of mean RPKM of DEGs related to cardiomyocyte (CM) structure and function (**f**), and extracellular matrix (ECM) and cardiac fibrosis (**g**). **h-i** Predicted upstream regulators of DEGs from Ingenuity Pathway Analysis (IPA).

To understand the role of ADAMTSL3 in response to LV pressure overload, we performed RNA-seq analysis on LVs one week post-AB, when both genotypes showed cardiac hypertrophy, but only L3-KO hearts were dilated (Fig. 2). A total of 233 differentially expressed genes (DEGs), 138 upregulated and 95 downregulated, were identified in L3-KO LVs with a false discovery rate (FDR) < 0.05 (Supplementary Data 1). 21 Kyoto Encyclopedia of Genes and Genomes (KEGG) pathways were identified from the DEGs, and the 10 most enriched included cardiomyopathies, CM function and cell-ECM interactions (Fig. 3a). Gene ontology (GO) analyses revealed enrichment of 110 biological pathways, 50 molecular function, and 51 cellular component categories, and of the 10 most enriched, several related to cardiac contractility and remodelling, cell-ECM interactions and TGFβ-signalling (Fig. 3b–d). Thus, unbiased RNA-seq analysis revealed the enrichment of pathways in line with exacerbated cardiac dysfunction and an ECM regulatory role of ADAMTSL3. Importantly, the most upregulated genes in L3-KO hearts included *Nppa* and *Myh7*, in line with cardiomyocyte remodelling and dysfunction^23^, and type I collagen (*Col1a1*) and periostin (*Postn*), whose levels are responsive to TGFβ signalling and indicative of increased fibrosis^24^ (Fig. 3e–g). Ingenuity pathway analysis (IPA, Qiagen) predicted MEF2C and TGFβ as major upstream regulators (USRs) of the CM- and CFB-related DEGs, respectively (Fig. 3h-i, Supplementary Data 1).

### ADAMTSL3 inhibits cardiac TGFβ signalling

**Fig. 4:**
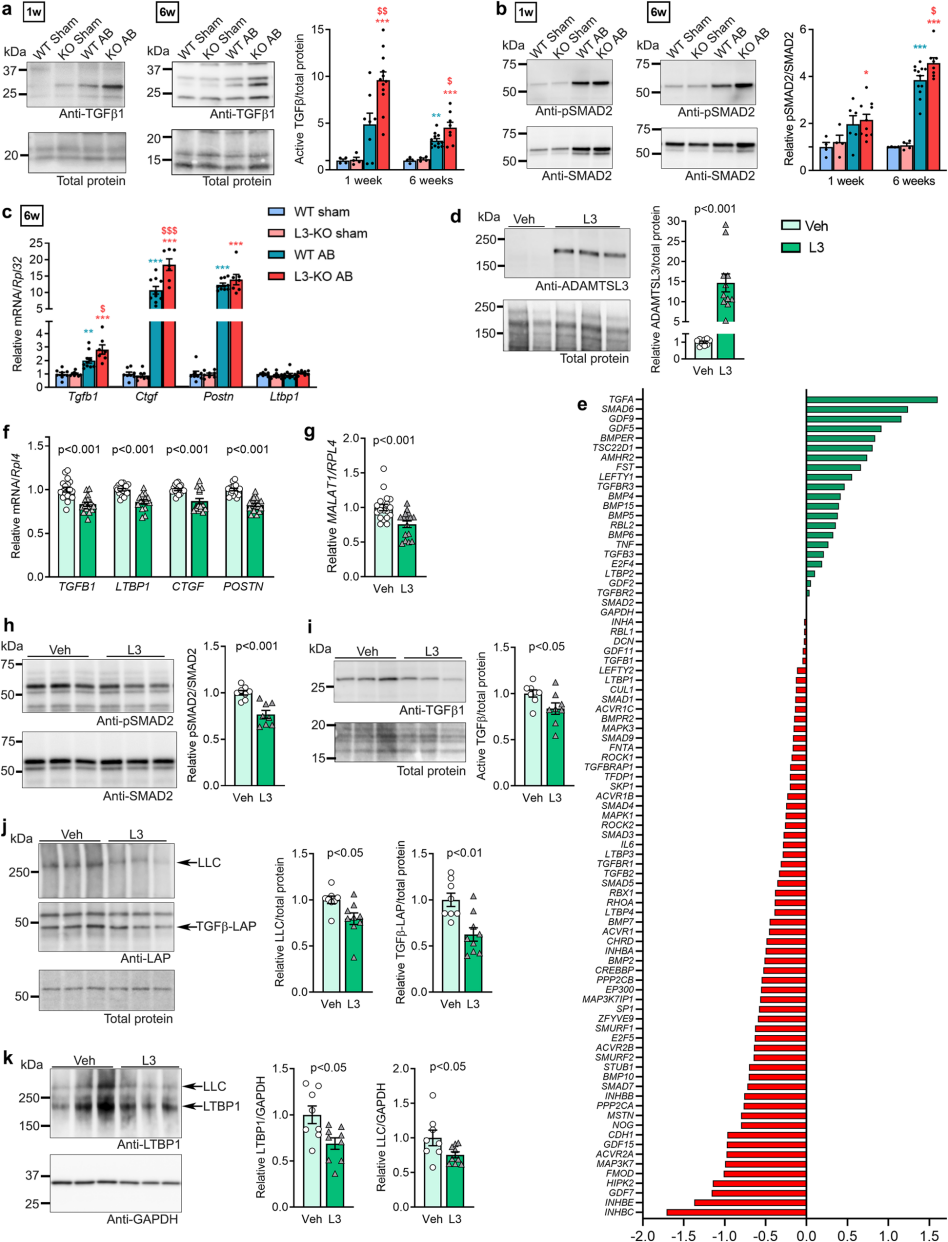
ADAMTSL3 modulates cardiac TGFβ signalling in response to increased LV afterload in vivo and in human cardiac fibroblasts in vitro. **a–c** Wild-type (WT) and *Adamtsl3* knock-out (L3-KO) mice were subjected to aortic banding (AB) or sham surgery for a total of 6 weeks. **a,b** Representative immunoblots and quantifications of active TGFβ1/total protein (**a**) and phosphorylated SMAD2 (pSMAD2)/total SMAD2 (b) in LV lysates, 1 week (sham: *n* = 4 WT and *n* = 4 KO, AB: *n* = 6-8 WT and *n* = 12 KO) and 6 weeks (sham: *n* = 4 WT and *n* = 4 KO, AB: *n* = 12 WT and *n* = 8 KO) post-AB. **c** mRNA/*Rpl32* levels of TGFβ (*Tgfb1*), connective tissue growth factor (*Ctgf*), periostin (*Postn*) and latent TGFβ binding protein 1 (*Ltbp1*) in AB mice vs sham 6 weeks post-AB (Sham: *n* = 8 WT and *n* = 8 KO, AB: *n* = 10 WT and *n* = 7 KO). Statistical analyses waere performed using the one-way ANOVA with Tukey’s multiple comparisons test, with *p < 0.05, **p < 0.01, and ***p < 0.001 for AB vs. sham, and ^$^*p* < 0.05, ^$$^*p* < 0.01, and ^$$$^*p* < 0.001 for L3-KO AB vs. WT AB. **d-k** Human foetal cardiac fibroblasts cultured for 7 days, producing a rich extracellular matrix (ECM) network, and transduced with ADAMTSL3 (L3) or control (vehicle, Veh) adenovirus on day 4. Data represent experiments from 3 different cell passages. **d** Representative immunoblot and quantification of ADAMTSL3/total protein in Veh (*n* = 9) and L3 (*n* = 11) lysates. **e** 62 downregulated and 22 upregulated genes in pooled L3 vs Veh mRNA samples (*n* = 18 in both groups) using an expression array of 84 TGFβ related genes, data are ΔΔCT values normalized to *GAPDH*. **f–g** mRNA/*RPL4* levels from qPCR of *TGFB1, LTBP1, CTGF, POSTN* (**f**) and *MALAT1* (**g**) in L3 (*n* = 16) vs Veh *(n* = 18). **h–k** Representative immunoblot and quantification of pSMAD2/SMAD2 (**h)**, active TGFβ1/total protein (i), latency associated protein (LAP)/total protein, including the large latent complex (LLC, > 250 kDa) (**j**), and latent TGFβ binding protein (LTBP1)/GAPDH, including the LLC (k), in L3 (*n* = 8-9) vs. Veh (*n* = 8) cell- and extracellular matrix lysates. Statistical analyses were performed using the Student’s *t*-test (**d, f-k**). All data (except **e**) are shown as individual value scatterplots with mean ± SEM.

As the bioinformatic analysis of RNA-seq data indicated increased TGFβ activity in L3-KO hearts one-week post-AB, we measured levels of active TGFβ and pSMAD2, a critical mediator of TGFβ signalling^5^, by immunoblotting. Levels of active TGFβ were higher in L3-KO hearts vs. WT at one-and six weeks post-AB (Fig. 4a). After one week of AB, pSMAD2 was increased in the L3-KO vs. sham, and after six weeks of AB, pSMAD2 was higher in L3-KO hearts vs. WT (Fig. 4b), thus indicating increased TGFβ signalling in L3-KO hearts. In line with this, L3-KO hearts showed increased expression of TGFβ (*Tgfb1*) and the downstream target of TGFβ signalling, connective tissue growth factor (*Ctgf*) at six weeks (Fig. 4c). *Postn* increased comparably in both AB groups, while *Ltbp1* was unchanged in all groups (Fig. 4c).

For further mechanistic insights, we overexpressed full-length ADAMTSL3 (L3) and a vehicle control adenovirus (veh) in cultures of human foetal CFBs (hfCFBs) (Fig. S5a), which form an extensive ECM network^11^. ADAMTSL3 overexpression was confirmed by increased mRNA (Fig. S5b) and protein (Fig. 4d). Using an expression array targeting 84 genes of the TGFβ signalling pathway on pooled L3 and Veh samples, we found 62 downregulated, and 22 upregulated genes in L3 (Fig. 4e). Several central mediators of canonical TGFβ signalling, e.g. *TGFB1, TGFB2, SMAD3, SMAD4, TGFBR1*, were downregulated, and the TGFβ signalling inhibitor SMAD6 was upregulated. Analysing individual L3 and Veh samples, we found that the genes encoding the components of the large latent TGFβ complex (LLC), i.e. *TGFB1* and *LTBP1*, the TGFβ signalling targets *CTGF* and *POSTN*, as well as the TGFβ signalling enhancer *MALAT1*^25^, were downregulated in L3 (Fig. 4f-g). Immunoblotting revealed reduced pSMAD (Fig. 4h) and reduced active TGFβ (Fig. 4i), indicating reduced TGFβ signalling. Additionally, levels of the LLC, consisting of the latency-associated peptide (LAP), transcribed from the *TGFB1* gene, and LTBP1 were reduced in L3 cell and ECM lysates (Fig. 4j-k), indicating reduced TGFβ production. Taken together, the loss-of-function and gain-of-function studies suggest ADAMTSL3 as an inhibitor of TGFβ signalling in the heart in vivo and in cardiac fibroblasts in vitro.

### ADAMTSL3 regulates deposition of insoluble collagen in the heart, and production of ECM molecules by cardiac fibroblasts

Tissue phase mapping (TPM) cardiac MRI was performed to evaluate myocardial function. Both genotypes developed reduced longitudinal and circumferential peak strain post-AB, suggesting systolic dysfunction (Fig. S6a-b). Furthermore, both genotypes developed reduced early diastolic strain rate (SRe), indicating less effective relaxation (Fig. S6c-d), and at three weeks, L3-KO showed reduced LV circumferential SRe compared to WT (Fig. S6d). Doppler echocardiography supported relative impairment of cardiac function in L3-KO compared to WT, as peak mitral inflow velocity (E) (Fig. S6e) and mitral valve inflow deceleration (MVD) (Fig. S6f) were reduced in the L3-KO. The reduced SRe in the L3-KO, may reflect LV stiffness, which can be related to myocardial fibrosis. As a next step therefore, we evaluated effects of ADAMTSL3 on ECM gene expression and biosynthesis.

**Fig. 5:**
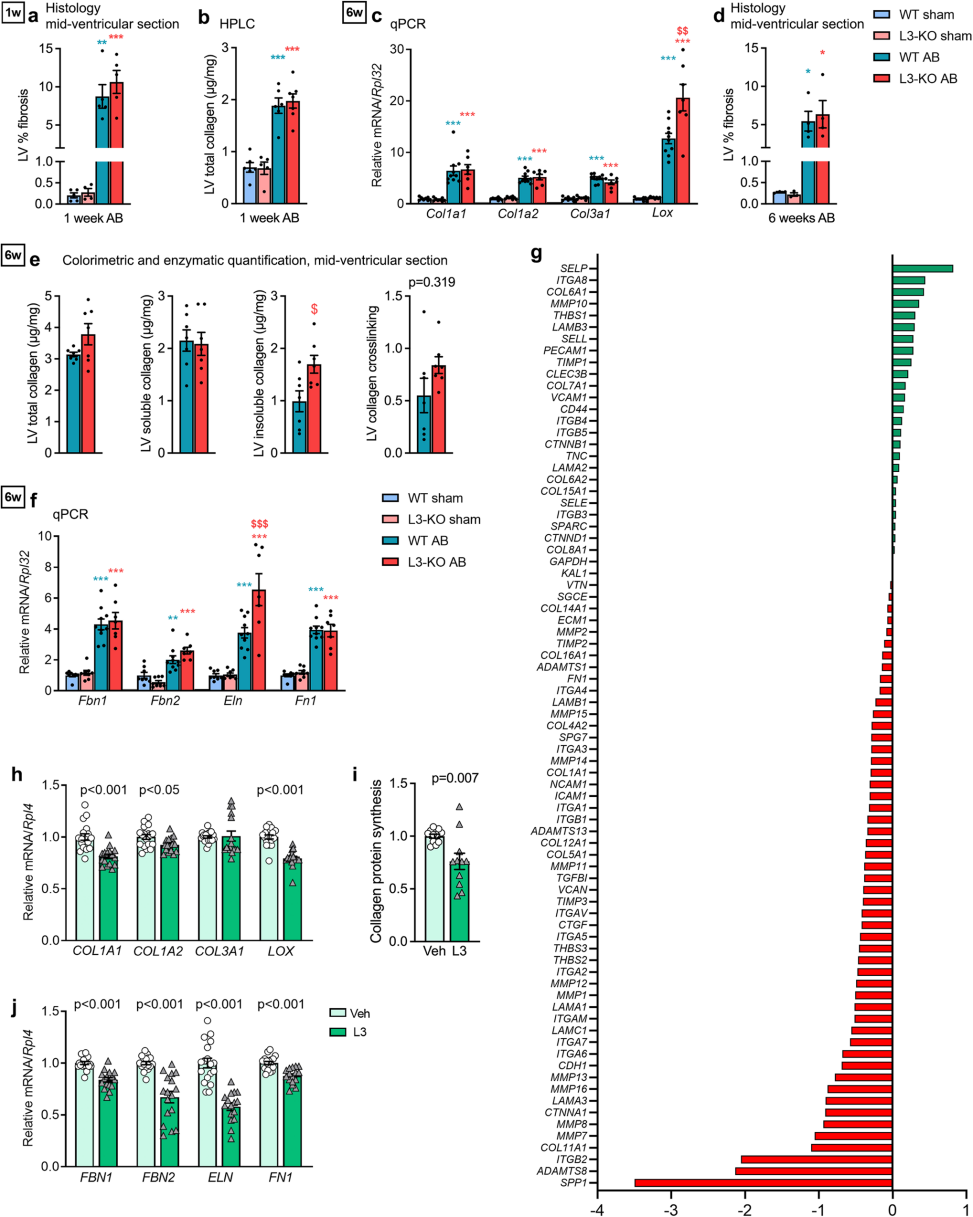
ADAMTSL3 regulates deposition of insoluble collagen in the heart, and production of ECM molecules by cardiac fibroblasts in vitro. **a–f** Wild-type (WT) and *Adamtsl3* knock-out (L3-KO) mice were subjected to aortic banding (AB) or sham surgery for 1 and 6 weeks. **a** Left ventricle (LV) % total collagen, calculated from Picrosirius Red, Fast Green, and Alcian Blue (RGB)-stained histology mid-ventricular sections at 1 week post-AB (Sham: *n* = 5 WT and *n* = 5 KO, AB: *n* = 5 WT and *n* = 5 KO). **b** LV type-I collagen content, as measured by peak hydroxyproline HPLC, 1-week post-AB (Sham: *n* = 6 WT and *n* = 5 KO, AB: *n* = 6 WT and *n* = 7 KO). **c** LV mRNA/*Rpl32* levels of collagens type I (*Col1a1, Col1a2*) and type III (*Col3a1*) and lysyl oxidase (*Lox*) (Sham: *n* = 8 WT and *n* = 8 KO, AB: *n* = 10 WT and *n* = 7 KO). **d** LV % total collagen in mid-ventricular sections at 6 weeks post-AB (Sham: *n* = 3 WT and *n* = 3 KO, AB: *n* = 4 WT and *n* = 4 KO). **e** Levels of total collagen, soluble collagen, insoluble collagen and collagen crosslinking in LVs 6 weeks post-AB, using colorimetric and enzymatic procedures on mid-ventricular sections *(n* = 7 WT and *n* = 7 KO). **(f)** mRNA/*Rpl32* levels of fibrillin-1/2 (*Fbn1, Fbn2*), tropoelastin (*Eln1*) and fibronectin-1 (*Fn1*), in L3-KO vs WT (Sham: *n* = 8 WT an*d n* = 8 KO, AB: n = 10 WT and *n* = 7 KO). Statistical analyses were performed using the one-way ANOVA with Tukey’s multiple comparisons test (**a–d, f**) or the Student’s *t*-test (**e**). P-values are reported as exact p-values or *p < 0.05, **p < 0.01, and ***p < 0.001 for AB vs. sham, and ^$^*p* < 0.05, ^$*$*^*p* < 0.01, and ^$$$^*p* < 0.001 for L3-KO AB vs. WT AB. **g–h, j** Human foetal cardiac fibroblasts (hfCFBs) were cultured for 7 days, producing a rich extracellular matrix (ECM) network, and transduced with ADAMTSL3 (L3) or control (vehicle, Veh) adenovirus on day 4. Data represent experiments in 3 different cell passages. **g** ΔΔCT expression values, normalized to *GAPDH*, of 53 downregulated and 25 upregulated genes in pooled L3 vs Veh mRNA samples (n = 18 in both groups) using an expression array of 78 cell adhesion and ECM-related genes. **h** mRNA/*RPL4* levels from qPCR of *COL1A1, COL1A2, COL3A1, LOX* in L3 (*n* = 16) vs Veh (*n* = 18). **i** Collagen protein synthesis, as measured by radioactive decay (counts per minute) of [^3^H]-proline, incorporated over 48 h in L3 and Veh CFBs, isolated from 1-3 day-old rats (*n* = 3 isolations with *n* = 60 hearts giving *n* = 3-4 technical replicates per isolation). **j** mRNA/*RPL4* levels from qPCR of *FBN1, FBN2, ELN* and *FN1* in L3 (*n* = 16) vs Veh (*n* = 18) hfCFBs. Statistical analysis was performed using the Student’s *t*-test (h-j). All data (except g) are shown as individual value scatterplots with mean ± SEM.

In the RNA-seq data, we found upregulated *Col1a1* in L3-KO vs WT at one-week post-AB (Fig. 3g), suggesting increased collagen production at this time-point. We therefore sought evidence of fibrosis in mid-ventricular sections of WT and L3-KO mice. One-week post-AB, trichrome staining showed comparable collagen levels in both genotypes, i.e., 9% collagen in WT and 11% collagen L3-KO vs. 0.2-0.3% in sham controls (Fig. 5a and representative images in Fig. S7a). We also quantified collagen I protein content by hydroxyproline levels in LVs one-week post-AB, which, in line with the histology, indicated a similar increase in collagen content in L3-KO and WT after AB vs. sham (Fig. 5b). At six weeks post-AB, collagen I (*Col1a1* and *Col1a2)* and collagen III (*Col3a1*) mRNA were increased to a similar extent in both genotypes, as assessed with qPCR (Fig. 5c). Likewise, trichrome staining of mid-ventricular sections was comparable in both genotypes, i.e., 5.5% collagen in WT and 6.4% L3-KO at six weeks post-AB (Fig. 5d and representative images in Fig. S7b). Thus, no difference was found in tissue collagen amount between genotypes at the two-time points investigated post-AB.

Lysyl oxidase (*Lox)* mRNA was increased 1.7-fold in L3-KO compared to WT at six weeks post-AB (Fig. 5c), which could indicate increased collagen crosslinking in the L3-KO. We therefore analysed total, soluble and insoluble collagen content at six weeks post-AB using colorimetric and enzymatic assays on mid-ventricular sections. Importantly, L3-KO LVs had higher levels of insoluble collagen vs. WT (Fig. 5e). The difference in calculated collagen crosslinking (ratio between insoluble and soluble collagen), however, did not reach significance (p = 0.139) between genotypes (Fig. 5e).

To examine whether ADAMTSL3 regulates ECM expression in vitro, we used an expression array targeting 78 ECM and cell adhesion genes, and found 53 downregulated and 25 upregulated genes in pooled samples of L3 vs. Veh. Several cell-adhesion genes were upregulated, e.g. *SELL, SELP, PECAM1* and *VCAM1*. In line with increased expression in L3-KO AB hearts, *COL1A1, ITGB1* and *CTGF* were downregulated in L3, as were several secreted and cell surface matrix metalloproteinases (MMPs) and ADAMTS proteases (Fig. 5g). Reduced *COL1A1* expression was confirmed in individual hfCFB samples, whereas *COL3A1* was unchanged (Fig. 5h). Collagen protein production was further investigated in L3 vs Veh nrCFBs with a developing ECM (Fig. S5c-d), using radiolabelled proline incorporation. Importantly, we found that L3 cultures incorporated 25% less radio-proline than Veh (Fig. 5i), suggesting that ADAMTSL3 inhibits cardiac fibroblast collagen protein levels. Consistent with increased *Lox* expression in L3-KO hearts (Fig. 5c), L3 overexpression in hfCFB reduced *LOX* mRNA (Fig. 5h), suggesting that ADAMTSL may inhibit collagen crosslinking. In L3-KO hearts six weeks post-AB, elastin (*Eln*) expression was 1.8-fold higher than Veh, while expression of fibrillins (*Fbn1/2*) and fibronectin (*Fn1*) was unchanged (Fig. 5f). Finally, microfibril expression was analysed in hfCFBs cultures producing an extensive microfibril network^11^, and showed reduced expression of *FBN1, FBN2, ELN* and *FN1* in L3 vs. Veh (Fig. 5j). Notably, fibronectin fibrils support the assembly of collagen I, fibrillin microfibrils, elastin and LTBP-1 into the ECM^26^. Taken together, our data demonstrate that ADAMTSL3 affects collagen synthesis and crosslinking, as well as synthesis of other structural ECM molecules in hfCFB cultures, and in response to pressure overload.

### ADAMTSL3 regulates cardiac myofibroblast differentiation

**Fig. 6:**
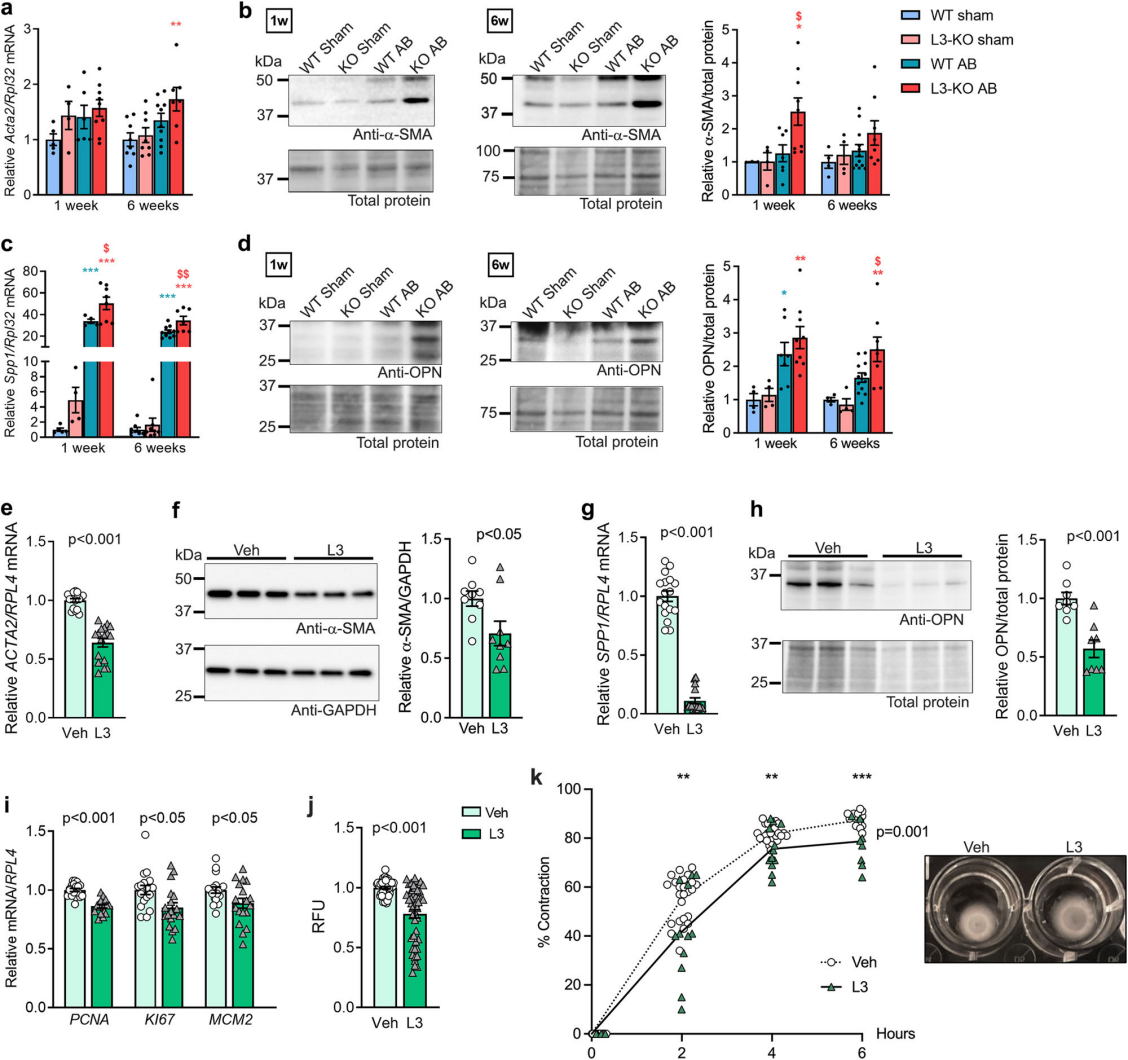
ADAMTSL3 regulates cardiac myofibroblast differentiation in vivo and in vitro. **a–d** Wild-type (WT) and *Adamtsl3* knock-out (L3-KO) mice were subjected to aortic banding (AB) or sham surgery for 1 and 6 weeks. **a** mRNA/*Rpl32* levels of α-smooth muscle actin (α-SMA, *Acta2*). **b** Representative immunoblots and quantification of α-SMA in LV protein extracts of WT and L3-KO 1 and 6 weeks post-AB or sham surgery. **c** mRNA/*Rpl32* levels of osteopontin (OPN, *Spp1*). **d** Representative immunoblots and quantification of OPN in LV protein extracts of WT and L3-KO 1 and 6 weeks post-AB or sham surgery. At 1-week post-AB, *n* = 4-5 WT and *n* = 4 KO sham, and *n* *=* 6–7 WT and *n* = 8–9 KO AB. At 6 weeks post-AB, *n* = 4-8 WT and *n* = 4-8 KO sham, and *n* = 10-12 WT and *n* = 7-8 KO AB. Statistical analyses were performed using the one-way ANOVA with Tukey’s multiple comparisons test (a-d). P-values are reported as *p < 0.05, **p < 0.01, and ***p < 0.001 for AB vs. sham, and ^$^*p* < 0.05, ^$$^*p* < 0.01, and ^$$$^*p* < 0.001 for L3-KO AB vs. WT AB. **e**–**k** Human foetal cardiac fibroblasts (hfCFBs) were cultured for 7 days, producing a rich extracellular matrix (ECM) network, and transduced with ADAMTSL3 (L3) or control (vehicle, Veh) adenovirus on day 4. Experiments were performed at 3 different cell passages. **e** *ACTA2*/*RPL4* levels in L3 (*n* = 16) vs Veh (*n* = 18). **f** Representative immunoblot and quantification of α-SMA/GAPDH in L3 (*n* = 8) vs Veh (*n* = 8). **g** *SPP1*/*RPL4* levels in L3 (*n* = 16) vs Veh (*n* = 18). **f** Representative immunoblot and quantification of OPN/total protein in L3 (*n* = 8) vs Veh (*n* = 8). **i** mRNA levels/*RPL4* of proliferating cell nuclear antigen (*PCNA*), marker of proliferation Ki-67 (*KI67*) and mini-chromosome maintenance complex component 2 (*MCM2*) in L3 (*n* = 18) vs Veh (*n* = 18). **j** Relative fluorescence units (RFU) reflecting EdU incorporation, as a measure of proliferation, in L3 vs Veh *(n* = 48). Data are given with mean ± SEM (**a**–**j**). **k** Collagen gel contraction of hfCFBs, as % contraction of initial gel area, at 2, 4 and 6 h, in L3 (*n* = 12) vs Veh (*n* = 24). Statistical analysis was performed using the Student’s *t*-test (**p**–**k**), with p-values reported as numerical values (**e**–**j**) or **p < 0.01 and ***p < 0.001, and the two-way repeated measures ANOVA with the Geisser-Greenhouse correction for comparison of multiple time-points (k, exact *p*-value shown in graph).

Osteopontin (*SPP1*) is essential for myofibroblast differentiation, and was identified as the most downregulated molecule in the expression array of L3 vs. Veh (Fig. 5g), and thus, we examined myofibroblast differentiation in WT and L3-KO hearts. We found comparable expression levels of the myofibroblast signature marker α-SMA (*Acta2*) between groups at one week and six weeks post-AB (Fig. 6a), but immunoblotting for α-SMA protein revealed a 2.5-fold increase in L3-KO vs. WT one-week post-AB (Fig. 6b), indicating increased myofibroblast differentiation. *SPP1* expression was increased in both genotypes post-AB, with higher levels in L3-KO hearts vs. WT (Fig. 6c), which was further confirmed at the protein level (Fig. 6d). *ACTA2* expression was reduced by 40% in L3 hfCFB cultures compared to Veh (Fig. 6e), and α-SMA protein was reduced by 25% (Fig. 6f). We confirmed downregulated *SPP1* in individual L3 hfCFB samples to 10% that of Veh levels (Fig. 6g), and a 40% reduction in osteopontin protein compared to Veh (Fig. 6h), indicating reduced myofibroblast differentiation. Next, pro-fibrotic hallmarks of activated CFBs, i.e., proliferation and the acquired ability to contract ECM^6^, were investigated, revealing reduced expression of the proliferative cell markers: proliferative cell nuclear antigen (*PCNA*), Ki-67 (*KI67*) and mini-chromosome maintenance complex component 2 (*MCM2*) in L3 (Fig. 6i), as well as reduced EdU incorporation (Fig. 6j). Most significantly, ADAMTSL3 overexpressing cells showed reduced ability to contract collagen gels (Fig. 6k), providing a functional readout of impaired myofibroblast differentiation.

### ADAMTSL3 did not have a direct effect on cardiomyocyte function

In line with reduced contractility in L3-KO hearts post-AB, RNA-seq revealed dysregulated CM sarcomere and contractility genes, e.g., the ryanodine receptor (*Ryr2*), titin (*Ttn*), sodium-calcium exchanger (NCX, *Slc8a1*), SERCA (*Atp2a2*) and phospholamban (*Pln*) (Fig. 3e–g). Thus, we investigated isolated CMs from adult WT and L3-KO mice. CMs were of similar size between genotypes (Fig. S8a), with comparable resting sarcomere length (Fig. S8b). During electrical pacing at 1 Hz, CMs exhibited similar contraction magnitudes, with parallel cell shortening by isoproterenol challenge (Fig. S8c), and similar contraction (Fig. S8d) and relaxation (Fig. S8e) kinetics, suggesting normal CM development in L3-KO hearts. As the phenotype of isolated CMs was similar in WT and L3-KO, and *Adamtsl3* expression was not detected in CMs (Fig. 1i), the exacerbated contractile dysfunction observed in L3-KO post-AB was interpreted as secondary to altered ECM and CFB function.

## Discussion

In the present study, we investigated regulation of ADAMTSL3 in biopsies from patients with heart failure, and its role in cardiac remodelling after AB-induced LV pressure overload in L3-KO and WT littermate mice. ADAMTSL3 was upregulated in failing hearts of patients and mice after AB, and was mainly produced by CFBs. Importantly, L3-KO mice demonstrated exacerbated contractile dysfunction and LV dilatation, with higher mortality after LV pressure overload. Molecular analyses of L3-KO and WT hearts post-AB revealed increased TGFβ signalling, CFB activation and dysregulated ECM production, with increased levels of insoluble collagen in L3-KO. Overexpression of ADAMTSL3 in cultured CFBs inhibited TGFβ signalling, myofibroblast conversion, ECM expression and collagen synthesis. Collectively, our data suggest a cardioprotective role for ADAMTSL3 in the failing heart, through inhibition of TGFβ activity and myofibroblast differentiation, with consequences for the cardiac ECM, cardiac remodelling and function upon pathogenic stimuli.

ADAMTSL3 levels were increased in heart biopsies from patients with heart failure and our studies in mice demonstrate an important role of ADAMTSL3 for cardiac function and survival. After pressure overload of the LV, the L3-KO developed exacerbated contractile dysfunction and cardiac dilatation. RNA-seq of post-AB hearts revealed DEGs associated with cardiomyopathy, CM calcium handling and contractility in the L3-KO. Since we did not detect *Adamtsl3* expression in CMs, and no difference was seen in CM function between isolated L3-KO and WT CMs, we interpret the reduced cardiac function in L3-KOs as secondary to altered ECM regulation by CFBs.

At baseline, L3-KO mice were viable and had normal body weight with no apparent phenotype, except for slightly longer limbs. Although baseline cardiac function or dimensions, as measured by echocardiography and MRI, were not affected by *Adamtsl3* inactivation, hearts from non-stressed L3-KO mice had increased weight. This indicates that interpretation of the post-AB cardiac phenotype should be done with caution, as the L3-KO hearts may be pre-disposed to exacerbated disease in response to a stressor.

Importantly, our study directly links ADAMTSL3 to cardiac function. This link was previously suggested, as *ADAMTSL3* is part of a rare syndrome known as Tetrasomy 15q25, arising from an inverted duplication of the distal chromosome 15^27,28^. Patient characteristics include growth abnormalities and complex cardiac malformations associated with high mortality^28^. The phenotypic cardiac defects only appear when *ADAMTSL3* is part of the chromosomal duplication^28^. Cardiac defects are also reported in patients with a heterozygous chromosomal microdeletion of the same region, including *ADAMTSL3*^29,30^.

The functional relationship between members of the ADAMTSL family and fibrillin microfibrils^13,15,17^ suggests ADAMTSLs as regulators of TGFβ, a function formerly demonstrated for ADAMTSL2^11,31^ and ADAMTSL6^14^. Our study adds to the growing body of evidence implicating ADAMTSL proteins in TGFβ regulation, as we here show increased levels and activity of TGFβ in L3-KO hearts post-AB, and inhibition of TGFβ in cultured CFBs overexpressing ADAMTSL3. As TGFβ is a major regulator of CFB activation and pro-fibrotic ECM production in the failing heart^7,32^, this may suggest ADAMTSL3 as a negative regulator of fibrotic and pathogenic signalling in the heart. ADAMTSL3 regulated CFB activation in mouse hearts as well as in cultured human CFBs, shown by altered levels of the myofibroblast markers α-SMA and osteopontin, alongside impaired CFB contraction in response to ADAMTSL3 overexpression. In cultured human CFBs, ADAMTSL3 overexpression resulted in altered expression of ECM molecules, including collagen. Although we did not see changes in total collagen in hearts in vivo, *Adamtsl3* inactivation resulted in altered ECM quality in the myocardium, with increased levels of insoluble collagen, that we speculate could be attributed to enhanced expression of *Lox*. Recent evidence suggest that the failing heart contains distinctive populations of activated CFBs, which hold varying characteristics, such as highly contractile, or highly collagen-producing^4,33^. We speculate that ADAMTSL3 may affect differentiation of specific myofibroblast populations that remodel the myocardium in ways other than mere collagen production. Indeed, over 30 dysregulated ECM genes have been identified in activated myofibroblasts from injured hearts^34^. After ADAMTSL3 overexpression in CFBs, we found reduced expression of *FBN1, FBN2, FN1*, and *ELN*, suggesting regulation of the ECM proteins that sequester the latent TGFβ complex. *Eln* expression was correspondingly increased in the L3-KO hearts.

TGFβ has long been attractive as a potential therapeutic target in heart disease, however, clinical translation is limited by adverse effects^32^, and alternative strategies for TGFβ pathway inhibition are warranted^35^. Furthermore, myofibroblast differentiation has been shown to be reversible in the failing heart^35^, and may be an important treatment target. Whether ADAMTSL3 holds therapeutic potential for inhibition of myofibroblast differentiation remains to be shown. Mechanistically, ADAMTSL3 binds to fibrillin-1 and LTBP1^13^, indicating a potential mechanism of regulating TGFβ activity. We suggest that ADAMTSL3 restricts TGFβ bioavailability in the ECM, possibly in conjunction with ADAMTSL2^11^. We further propose IFN-γ, a negative regulator of TGFβ and pro-fibrotic signalling in the heart^36,37^, as an upstream regulator of ADAMTSL3.

In conclusion, the increased mortality and exacerbated cardiac phenotype of L3-KO mice after pressure overload suggests a cardioprotective role for ADAMTSL3 in the failing heart. Mechanistically, our results indicate that ADAMTSL3 supports cardiac function via limitation of TGFβ activation and myofibroblast differentiation. In future investigation, this possibility could be addressed by increasing ADAMTSL3 levels experimentally in models of heart disease, to reveal potential cardioprotective effects.

## Methods

### Ethics

The use of human cardiac biopsies was approved by the Regional Committee for Medical Research Ethics (REK ID 07482a, and 2010/2226) the South-Eastern Regional Health Authority, Norway, and is in accordance with the Declaration of Helsinki. All patients, or the next of kin of donor controls, signed a written informed consent. The biopsies were obtained at Oslo University Hospital (OUH), and all patients received standard clinical evaluation, treatment and follow-up in accordance with hospital guidelines. The mouse models and experiments were approved by the Cleveland Clinic IACUC (protocol number 2020-2450) and Norwegian National Animal Research Committee (approval ID 16614) and were in accordance with the NIH Guide for the Care and Use of Laboratory Animals, as well as the ARRIVE guidelines for reporting animal research.

### Human heart tissue samples

From patients with symptomatic AS, LV free wall biopsies (*n* = 11) were taken during open-heart surgery for aortic valve replacement. Control LV biopsies (*n* = 11) were taken from normally contracting tissue of patients undergoing surgery for coronary artery disease. Patient characteristics were previously described^18^. In brief, all patients had EF > 50%. AS patients had non-dilated LV (LVIDd 4.81 ± 0.23 cm) and hypertrophic LV walls (IVSd 1.26 ± 0.06 cm and LVPWd 1.22 ± 0.07 cm). Due to limited sample material, only RNA was isolated from this cohort.

LV biopsies from patients with secondary, ischemic DCM (*n* = 20) were obtained from beating hearts immediately after explantation. LV tissue from non-diseased hearts considered for transplantation, but rejected due to surgical reasons, served as controls (*n* = 3 RNA and *n* = 7 protein samples). Patient and donor characteristics were described previously^19^. In brief, hearts were dilated (LVIDd 7.41 ± 0.22 cm), had reduced systolic function (LVEF of 19.2 ± 1.6%) and walls were not hypertrophic (IVSd 0.81 ± 0.05 cm and LVPWd 0.71 ± 0.02 cm, respectively). Tissue samples were snap-frozen in liquid nitrogen and stored at −70 °C.

### CRISPR-Cas9 targeting of *Adamtsl3* in mice

A new *Adamtsl3* mutant allele was generated in C56BL/6 J mice using CRISPR/Cas9. A guide RNA (gRNA) with sequence 5’ CAGCCACCAGGATCCTGTCC 3’ (protospacer adjacent motif (PAM) sequence underlined) was selected for targeting exon 2 (the signal peptide) of the *Adamtsl3* gene (ENSMUST00000173828.2). gRNA was transcribed in vitro by Applied StemCell Inc (Milpitas, CA, USA) to generate single gRNA molecules. Sanger sequencing of the PCR-amplified targeted region indicated 62.5% activity. Accordingly, this gRNA along with Cas9 protein was injected into C57BL/6 J embryos which were transferred to surrogate CD1 dams and analysed by subsequent PCR amplification of the targeted region using genomic DNA and sequence analysis. A founder mouse with a Sanger sequence-validated 5 bp deletion, with germ-line transmission of the mutant allele, was retained and crossed into the C57BL/6 J strain. WT and L3-KO ear biopsies were genotyped using the WT forward primer: 5’-GGCTTCCTGGACAGGATCC-3’, or the L3-KO forward primer: 5’-CCCATGGCTTCCTGGATCC-3’, and a common reverse primer: 5’-GGGTGTGTTAACAGTGAATCC-3’, in two separate PCR reactions, with resulting PCR products of 428 bp (Fig. S3a). Ablated expression of *Adamtsl3* in L3-KO hearts was confirmed by RNA-seq (Fig. S3b) and RT-qPCR (Fig. S3c) of LV tissue.

### Mouse model of cardiac pressure overload

Cardiac pressure overload from increased LV afterload was induced in 8 to 11-week-old L3-KO and WT male littermates by an experienced mouse surgeon blinded to genotype. High-precision banding of the ascending aorta (AB) was performed using a an improved procedure, as recently described, with nitrile O-rings of a fixed inner diameter (0.55 mm)^20^ rather than a suture, resulting in reproducible blood flow restriction, low post-operative mortality and consistent cardiac phenotypes. The mice were anesthetized by breathing oxygen with 4% isoflurane in a chamber and intubated and ventilated breathing 2% isoflurane during AB or sham surgery. Subcutaneous injections of 0.3 mg/mL buprenorphine (0.1 mg/kg) were given before and after surgery. Additional analgesics were given to animals showing any sign of pain over the following 24 h.

Echocardiography was performed at baseline, and one, three and six weeks post-AB. Animals were anesthetized in a chamber with 4% isoflurane and anaesthesia was maintained breathing 1-2% isoflurane through a mask. Cardiac dimensions were captured by a blinded, experienced operator using the VEVO 2100 imaging system (VisualSonics, Toronto, Canada). Analysis of echocardiography images was performed blinded to genotype using the Vevo LAB ultrasound analysis software (VisualSonics).

Magnetic resonance imaging (MRI) was performed at baseline, and one and three weeks post-AB. Animals were anesthetized in a chamber with 4% isoflurane, and the anaesthesia was maintained on 1-2% isoflurane. Acquisitions were prospectively respiration gated and R-peak triggered. Electrocardiogram (ECG), respiration frequency, and body temperature were monitored during the examination. The MRI acquisition was performed using a 9.4 T magnet (Bruker, USA) with a 35 mm Rapid QUAD radio frequency coil. One 4-chamber long-axis CINE sequence slice as well as a stack of short-axis slices covering the LV was acquired. The imaging parameters were: echocardiography time =1.7 ms (long-axis) and 2.05 ms (short-axis), repetition time =5.0 ms (long-axis) and 4.7 ms (short-axis), field of view =25 mm×25 mm, matrix =128 pixels x 128 pixels, slice thickness =1.0 mm, flip angle 15°, signal averaging =3 times, total acquisition duration =1-2 min (long-axis) and 6-10 min (short-axis). We acquired one 4-chamber long axis and one mid-ventricular short axis tissue phase mapping (TPM) recording using compressed sensing^38^. TPM parameters were: echocardiography time =1.7 ms, repetition time =3.5 ms, field of view =25 mm×25 mm, matrix =96×24 pixels (4x under-sampling, 96×96 after reconstruction), slice thickness =1 mm, flip angle =10°, for a total acquisition time of approximately 1.5-3 min per slice, depending on the heart rate. Analysis was performed blinded using in house MATLAB scripts as previously described^39^.

Animals were anesthetized in a chamber with 5% isoflurane, and beating hearts and lungs were harvested from animals under deep terminal anaesthesia breathing 5% isoflurane through a mask. The LVs were excised, rinsed in PBS, snap-frozen in liquid nitrogen and stored at −70 °C.

### RNA in-situ hybridization

Hearts from AB- or sham-operated mice were fixed in 4% paraformaldehyde (PFA) and embedded in paraffin. 7 μm paraffin sections were cut and *Adamtsl3* mRNA was detected using in-situ hybridization with the RNAscope technology (Advanced Cell Diagnostics, Cat. No. 465521). A HybEZ oven was used for hybridization and the 2.5 HD Red detection kit was used for visualization. The tissue was counterstained with hematoxylin. An Olympus BX51 microscope (Olympus, Center Valley, PA) with a Leica DFC7000T camera was used for imaging with the Leica Application Suite v4.6 software.

### Histology

Snap-frozen LV tissue was fixed in 4% PFA and embedded in paraffin. Mid-ventricular sections of 7 µm thickness were taken on Superfrost Plus glass slides (Fisher) using a Leica RM2255 microtome. These slides were then stained with Picrosirius Red, Fast Green, and Alcian Blue (RGB) trichrome stain as preciously described^40^, to visualize collagen protein in the sections. Images were obtained using an Olympus BX51 upright microscope with a Leica DFC7000T camera and Leica Application Suite v4.6 imaging software. Areas with collagen, based on colour thresholds, were detected automatically from RGB trichrome images by a custom Fiji ImageJ 1.53c (NIH, USA) macro, which calculated percent fibrosis. The same macro was applied to all images from both genotypes at both timepoints.

### High-performance liquid chromatography

Quantitative analysis of hydroxyproline in mouse LVs, a measure of collagen protein content, was performed using high-performance liquid chromatography (HPLC) with the AccQ-Fluor kit (Cat # WAT052880, Waters, MA, USA) according to manufacturer’s protocol. 15 mg tissue was homogenized in liquid nitrogen, hydrolysed overnight in 6 M HCl at 112 °C, dried and derivatized with the AccQ-Fluor borate buffer (Waters). The derivatives were separated using reversed-phase HPLC with the Ultimate 3000 system (Nerliens Meszansky, Oslo, Norway), and quantified by fluorescence detection, with hydroxyproline standards (Fluka, Buchs SG, Switzerland).

### Insoluble collagen

Insoluble collagen in mid-ventricular sections was measured using a colorimetric fast green/picrosirius red assay, by subtracting soluble collagen from total collagen. Total collagen was measured as previously described^41^, and an enzymatic Sircol-based assay (Biocolor, UK) was used to quantify soluble collagen^42^. Collagen crosslinking was calculated as the ratio of insoluble and soluble collagen. All measurements were performed in duplicate by an experienced researcher, blinded to genotype. The amount of collagen was normalized to total protein.

### Isolation and analysis of single cardiomyocytes

LV cardiomyocytes were isolated from WT and L3-KO mice as previously described^43^. In brief, freshly excised hearts were cannulated though the aorta on a constant flow Langendorff setup, perfused with isolation buffer containing (in mmol/L): 130 NaCl, 5.4 KCl, 25 HEPES, 0.5 MgCl_2_, 0.4 NaH_2_PO_4_ and 22 glucose, pH 7.40, 37 °C. After washing, hearts were digested by perfusion with 2.0 mg/mL type 2 collagenase (290 unit/mg, Worthington Biomedical Corp., Lakewood, NJ, USA) solution for 6 min. The LV was dissected and agitated with 0.2 mg DNase (Worthington) and 250 µL BSA (40 mg/mL). Cells were filtered through a 200 µm mesh and settled. The pellet was washed twice and Ca^2+^ levels were gradually increased to 0.2 mM. Isolated CMs were plated in a chamber on an inverted microscope (Observer D1, Zeiss, Germany), and continuously superfused with Hepes Tyrode buffer (37 °C) containing (in mmol/L): 140 NaCl, 5.4 KCl, 1.8 CaCl_2_, 0.5 MgCl_2_, 5.0 Hepes, 5.5 glucose, 0.4 NaH_2_PO_4_, pH 7.40. During recordings, cells were paced at 1 Hz. Sarcomere positions were recorded using a 63 x/1.2 W water objective (Objective C-Apochromat, Zeiss, Germany) and a high-speed camera (Digital CMOS camera C11440-22CU, Hamamatsu, Japan) at a frame rate of 2 ms. Individual sarcomere contractions were analyzed by Image J (NIH), Clampfit (Axon Instruments) and custom Python scripts. In brief, brightfield images were inverted and filtered by an FFT (Fast Fourier Transform) bandpass filter. A region containing 10-15 sarcomeres was selected. The intensity of each vertical pixel line was averaged and Z-line (peak value) positions were obtained by a fitting curve using parabolic functions. By subtracting neighbouring Z-line positions, single sarcomere contractions were obtained, and normalized to resting SL to obtain fractional shortening. Data was averaged across 10-15 sarcomeres in each cell, and across 3 consecutive contractions.

### Primary cultures of neonatal cardiomyocytes and cardiac fibroblasts

Neonatal rat primary CMs (nrCM) and CFBs (nrCFB) were isolated as previously described^44^. Briefly, beating hearts were harvested from 1-3 day old Wistar rats (Janvier Labs) after decapitation. The ventricles were excised and digested with a pancreatin/collagenase solution. The adherent cell population was isolated from non-adherent cells by 20 min attachment to uncoated culture flasks. According to microscopy and qPCR analyses^11^, these cells were mainly CFBs. CFBs were cultured for one week, then plated at 3.8 ×10^4^ cells/cm^2^ in six-well plates, and cultured for another week before harvest. The non-attached cell fraction, mainly CMs, but with presence of ECs^11^, was plated at 3.8 ×10^4^ cells/cm^2^ on six-well plates coated with gelatin and fibronectin. All cells were cultured in serum-containing Dulbecco’s Modified Eagle medium (Gibco), in a humidified incubator with 5% CO_2_ at 37˚C. ADAMTSL3 was overexpressed in neonatal rat CFBs using a replication-deficient human adenovirus serotype 5 (Ad5 dE1/E3) vector encoding *ADAMTSL3* (Genbank RefSeq BC128389) under the cytomegalovirus (CMV) promoter (L3), or a vehicle control (Ad5 dE1/E3-CMV-Null) (Veh) (both commercially available from Vector Biolabs, PA, USA). Cells were seeded and transduced after 24 h (day 1) in a 4-day culture protocol (Fig. S5a), in which cells deposit a developing, immature ECM network^11^. Transduction was performed in growth medium at a virus titer of 5 ×10^6^ plaque forming units (PFU), giving a multiplicity of infection (MOI) of 100. Growth medium was changed to serum-free medium 24 h after transduction. Successful overexpression was verified by increased *Adamtsl3* mRNA in L3 vs. Veh control cells (Fig. S5b). For stimulation with IFN-γ, cells were grown in serum-free medium for 24 h, and 5 ng/mL recombinant IFN-γ (Z03274, GenScript, NJ, USA) was added 3 h before harvest.

### Human cardiac fibroblast cultures

Human foetal cardiac fibroblasts (hfCFBs) were obtained from Cell Applications, Inc (Cat# 306-05 f) and cultured in Cardiac Fibroblast Growth medium (Cat# 316-500, Cell Applications) or Fibroblast Basal medium (Cat# 115-500, Cell Applications), both supplemented with 1% penicillin/streptomycin, in a 5% CO_2_ humidified incubator at 37˚C. Cells were plated at 10,000 cells/cm^2^ for experiments, unless otherwise stated. A unique hfCFB culture protocol was used, in which the cells develop a mature ECM, including components of the TGFβ system, as previously described^11^. Briefly, cells were cultured for a total of seven days, and transduced on day 4 with L3 or Veh at 5 ×10^6^ PFU. After 24 h, transduction medium was changed to serum-free basal medium (Fig. S5c). Successful overexpression was verified by increased *ADAMTSL3* mRNA in L3 vs. Veh control cells (Fig. S5d).

### Collagen gel contraction assay

The collagen gel contraction assay was performed essentially as described^11^. L3 or Veh transduced hfCFBs were trypsinized, centrifuged and resuspended in serum-free medium. Cells were mixed with a collagen solution containing 3 mg/mL bovine collagen I (Bovine PureCol®, Advanced BioMatrix), 2x DMEM (Merck Millipore) and 0.2 M HEPES (pH 8), and plated at 36.5 ×10^3^ cells/cm^2^ in 24-well plates, pre-coated with 2% BSA in PBS overnight at 37 °C. The collagen gels polymerized for 2 h at 37 °C, and serum-free medium was added to detach the gels. The circumference of the gels, corresponding to the amount of gel contraction, was measured 6 and 24 h later, using ImageJ 1.53c (NIH).

### [^3^H] Proline incorporation assay

nrCFBs were seeded in 12-well plates and transduced with Veh or L3 after 24 h. The medium was changed after 24 h to serum-free medium containing 50 µM/mL ascorbic acid and 1 µCi L-[2,3-^3^H]-Proline (Cat# NET323001 MC, PerkinElmer, Inc, MA). On day 4, cells were washed with cold PBS, lysed in 1M NaOH and diluted using the OptiPhase HiSafe 3 liquid scintillation cocktail (Cat# 1200.437, PerkinElmer). The amount of radiolabelled proline present in each sample, as a surrogate for collagen protein biosynthesis^45^, was measured as counts per minutes (CPM) using the Wallac Winspectral 1414 liquid scintillation counter (PerkinElmer).

### EdU incorporation assay

L3 or Veh transduced hfCFBs were plated at 1000 cells/mm^2^ in 96-well plates (Cat# 6005430, Perkin Elmer). Cells were labelled with 10 µM EdU for 2 h, following 24 h serum-starvation. Cells were fixed using the Click-iT™ EdU Proliferation Assay (Cat# C10499, CyQUANT, Invitrogen), and EdU fluorescence was measured on the Hidex Sense Microplate Reader (LabLogic Systems, Sheffield, UK), essentially as described^11^.

### Gene expression analysis

Total RNA was isolated from cardiac tissue or cultured cells with the RNeasy Mini Kit (Cat# 74106, Qiagen Nordic, Norway). The iScript cDNA Synthesis Kit (Cat# 1708891, Bio-Rad Laboratories, Inc., Hercules, CA) was used for reverse transcription and cDNA generation from isolated RNA. Relative gene expression in each sample was determined using TaqMan Gene Expression Assays (Table SIII) and premade TaqMan gene expression arrays for TGFB pathway (Cat# 4414097) and human extracellular matrix and adhesion molecules (Cat# 4414133), with TaqMan Universal PCR Master Mix (Cat# 4304437). The QuantStudio 3 Real-Time PCR System (Applied Biosystems, Foster City, CA) was used for PCR amplification and detection.

RNA sequencing (RNA-seq) was performed on isolated LV tissue from WT (*n* = 6) and L3-KO (*n* = 10) hearts one-week post-AB, similarly to previously described^46^. Briefly, total RNA was isolated using TRI Reagent (Sigma Aldrich) by phenol-chloroform extraction. LV tissue was homogenized in 1 mL TRI Reagent using the Tissuelyzer II (Qiagen). Ribosomal RNA was removed using the NEBNext® rRNA Depletion Kit (Human/Mouse/Rat, New England Biolabs). Total stranded RNA-seq libraries were generated using the CORALL Total RNA-Seq Library Prep Kit (Lexogen), with AMPure beads (Beckman Coulter) for purification. RNA-seq libraries quality controlled using the 2100 Bioanalyzer (Agilent). QC and sequencing was carried out at Babraham Institute Next Generation Sequencing facility, Cambridge, UK. RNA-seq libraries were sequenced on one lane of the HiSeq2500 (Illumina) with the HiSeq2500-RapidRun 100 bp Single End sequencing run. RNA-seq data were aligned using HiSAT2^47^ to the *Mus musculus* reference genome GRCm38/mm10. Reads were trimmed prior to alignment using Trim Galore, with a Phred quality score for base calling cutoff of 20, corresponding to a maximum error of 1 in 100 bases, and with a maximum trimming error rate of 0.1. Trimmed and aligned sequence files were imported as BAM files into SeqMonk (v1.42.0) for visualization and analysis. RNA-seq reads were quantified by read count quantitation and global normalisation performed to total read count for each library and expressed as reads per kilobase million (RPKM). Analysis for differentially expressed genes (DEGs) was performed using the R-based software DESeq2^48^. Raw (non-log transformed) read counts were used as input and global normalization performed to total library size.

### Gene expression data mined from available online databases

Mouse *Adamtsl3* cell expression data was obtained from the Single Cell Expression Atlas database (https://www.ebi.ac.uk/gxa/sc/home), EMBL-EBI, Cambridgeshire, UK, accessed on 31.05.2022)^22^. Human *ADAMTSL3* expression data from the LAA and LV of deceased organ donors was obtained from the GTEx Project Portal (https://gtexportal.org/home), Broad Institute, Boston, MA, accessed on 31.05.2021). The charts were modified from the interactive graphics functions of the databases.

### Immunoblotting

Proteins were extracted from human and mouse cardiac tissue according to a previously described protocol^20,44^, with a PBS‐based lysis buffer containing Triton X‐100 (1%), Tween‐20 (0.1%), protease inhibitors (cOmplete EDTA‐free Mini, Roche Diagnostics) and PhosStop phosphatase inhibitors (Roche Diagnostics). Proteins from cell cultures were extracted using a lysis buffer containing SDS (1%) and Tris-HCl (31.5 mM, pH 6.8). Protein lysates were sonicated using the Branson Sonifier® S-150D (Emerson Electric Co. St. Louis, MO), centrifuged at 15.000 G for 20 min, and the supernatant was stored at -70 °C. SDS-PAGE and immunoblotting was performed using Criterion TGX gels and Trans-Blot Turbo polyvinylidene difluoride (PVDF) membranes (Bio-Rad) on the Trans-Blot Turbo semi-dry blotting system (Bio-Rad). The following antibodies were used: anti-phospho-Smad2 (Ser465/467, Cat # 3108, Cell Signaling), anti-Smad2/3 (Cat # 3102, 8685, Cell Signaling), both diluted 1:1000 in 5% milk, anti-TGFb (Ab92486, Abcam) 1:1000, 1% casein, anti-LAP (AF-246, R&D Systems) 1:1000 in 5% milk under non-reducing conditions, anti-LTBP1 (Cat # MAB-388, R&D Systems) diluted 1:1000 in 5% milk under non-reducing conditions, anti-α-SMA (Cat # A5228, Sigma) diluted 1:10,000 in 5% milk, anti-OPN (Ab181440, Abcam) 1:1000 in 1% milk, anti-GAPDH (Cat # sc-32233, Santa Cruz Biotechnology) diluted 1:1000 in 5% milk, anti-ADAMTSL3 (HPA034773, Atlas Antibodies) 1:1000 in 1% casein, and anti-ADAMTSL3 (in-house polyclonal antibody produced in rabbit) 1:1000 in 1% casein. Secondary antibodies for mouse or rabbit (1:2000, Santa Cruz Biotechnology) was used. ECL Prime Western Blotting Detection Reagent (Amersham) was used for secondary antibody detection and total protein was detected using Revert 700 protein staining (Licor). Fluorescence or chemiluminescence was imaged using the Azure 600 western blot imaging system (Azure Biosciences). Protein bands were quantified using ImageJ 1.53c (NIH). Full western blot membranes used for calculations are shown under Supplementary blots in the Supplementary Information.

### Statistics and reproducibility

Statistical differences were tested using Prism 8.3.0 (GraphPad). Data are presented with mean ± standard error of the mean (SEM). The distribution of the data was evaluated using the Shapiro-Wilk test. When comparing two normally distributed groups, the unpaired Student’s *t*-test was applied. When comparing multiple groups, one-way ANOVA with Tukey’s or Dunnett’s multiple comparisons tests was applied. For the comparison of multiple time-points, the two-way repeated measures ANOVA with the Geisser-Greenhouse correction was used. For comparison of survival curves, the Log-rank (Mantel-Cox) test was performed. *P* values <0.05 were considered statistically significant. *P*-values are reported as exact values, or, for mouse model data, significance is given as symbols representing *p < 0.05, **p < 0.01, and ***p < 0.001 for AB vs. sham, and ^$^*p* < 0.05, ^$$^*p* < 0.01, and ^$$$^*p* < 0.001 for L3-KO AB vs. WT AB. For RNA-seq data, RPKM values ≥1.0 were deemed to be above noise and genes with ≥ 1.0 in four or more samples were deemed as detectable in this dataset. Benjamini-Hochberg correction for multiple testing was used and FDR of 0.05. Functional enrichment analysis for GO terms and KEGG pathways was performed in Database for Annotation, Visualization and Integrated Discovery (DAVID) v6.8 analysis wizard^49^ (accessed on 27.08.21), and IPA from Qiagen was used for prediction of upstream regulators. All cell culture experiments were conducted minimum three times, representing three biological replicates, i.e., isolation rounds for primary cells or separate seeding timepoints for human foetal CFBs, each experiment with two or more technical replicates. The in vivo mouse experiments were conducted in three separate cohorts. Two cohorts were harvested at one week and one cohort was harvested at six weeks. The n of each experiment is given in the respective figure legends, with *n* = 3–12 in each group for each analysis.

### Reporting summary

Further information on research design is available in the Nature Research Reporting Summary linked to this article.

## Supplementary information

Supplementary Information

Description of Additional Supplementary Data

Supplementary Data 1

Supplementary Data 2

Nature reporting summary

## Data Availability

The RNA-seq data used to generate the plots and charts of Fig. 3 are uploaded in Supplementary Data 1, and was deposited in its entirety to the European Nucleotide Archive (ENA, EMBL-EBI, Wellcome Genome Campus, Hinxton, Cambridgeshire, UK), accession: PRJEB47017. All numerical source data used for generating the main figures are uploaded Supplementary Data 2. Unedited western blot images used to generate figures are presented under Supplementary Blots in the Supplementary Information. All other data are available from the corresponding author on reasonable request.
