# Supplementary material for: ADAMTSL3 knock-out mice develop cardiac dysfunction and dilatation with increased TGFβ signalling after pressure overload
Source: Commun Biol. 2022 Dec 20;5:1392. doi: 10.1038/s42003-022-04361-1 (PMC9767913; doi:10.1038/s42003-022-04361-1)
Supplement: Supplementary file 1 — Supplementary Information [file 42003_2022_4361_MOESM1_ESM.pdf]

Supplementary information

ADAMTSL3 knock-out mice develop cardiac dysfunction and dilatation with increased TGFβ signalling after pressure overload

Karoline B. Rypdal[1,2,3*], A. Olav Melleby[1,4], Emma L. Robinson[5], Jia Li[1], Sheryl Palmero[1], Deborah E. Seifert[6], Daniel Martin[6], Catelyn Clark[6], Begoña López[7,8], Kristine Andreassen[1], Christen P. Dahl[9], Ivar Sjaastad[1], Theis Tønnessen[1,10], Mathis K. Stokke[1], William E. Louch[1], Arantxa González[7,8], Stephane Heymans[5,11], Geir Christensen[1], Suneel S. Apte[6], Ida G. Lunde[1,2,3]

[1]Institute for Experimental Medical Research, Oslo University Hospital and University of Oslo, Oslo, Norway.
[2]Division of Diagnostics and Technology, Akershus University Hospital, Lørenskog, Norway.
[3]K.G. Jebsen Center for Cardiac Biomarkers, University of Oslo, Oslo, Norway.
[4]Department of Molecular Medicine, Institute of Basic Medical Sciences, University of Oslo, Oslo, Norway.
[5]Department of Cardiology, Maastricht University, CARIM School for Cardiovascular Diseases, Maastricht, Netherlands.
[6]Department of Biomedical Engineering, Cleveland Clinic Lerner Research Institute, Cleveland, OH USA.
[7]Program of Cardiovascular Diseases, CIMA Universidad de Navarra and IdiSNA, Pamplona, Spain.
[8]CIBERCV, Carlos III Institute of Health, Madrid, Spain.
[9]Department of Cardiology, Oslo University Hospital Rikshospitalet, Oslo, Norway.
[10]Department of Cardiothoracic Surgery, Oslo University Hospital Ullevål, Oslo, Norway.
[11]Centre for Molecular and Vascular Biology, Department of Cardiovascular Sciences, Leuven, Belgium.

*Corresponding author contact details:
Karoline Bjarnesdatter Rypdal, MSc, PhD
Institute for Experimental Medical Research (IEMR),
Oslo University Hospital Ullevål,
Building 7, 4th floor, Kirkeveien 166, 0407 Oslo, Norway.
Phone: +4791193598
e-mail: k.b.rypdal@medisin.uio.no

Supplementary figures

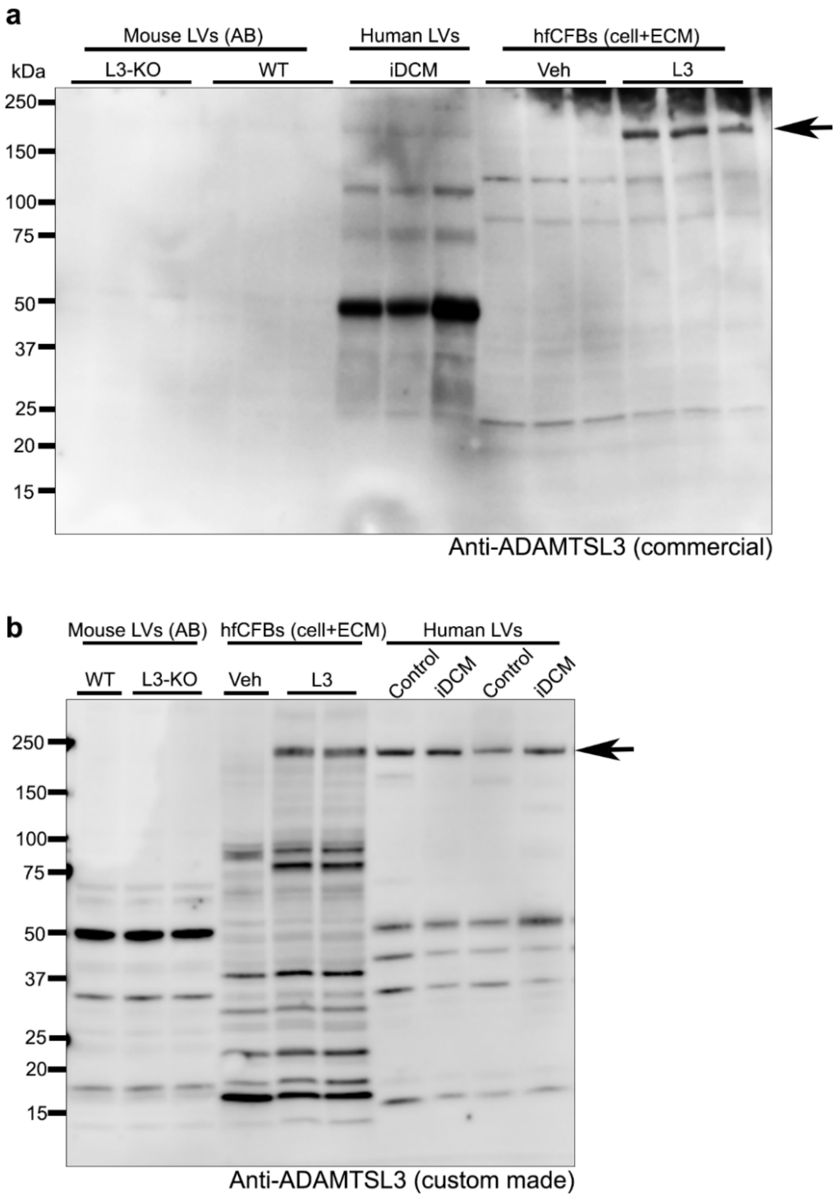

Fig. S1 ADAMTSL3 antibodies detect human, but not mouse ADAMTSL3
Representative immunoblots of ADAMTSL3 antibody validation in left ventricular (LV) tissue from mouse (after aortic banding (AB)) and human (control or ischemic dilated cardiomyopathy (iDCM)). ADAMTSL3 is an ECM glycoprotein with expected size of 210 kDa[1] (1691 amino acids). LV tissue from L3-KO mice was used as negative control and human foetal cardiac fibroblasts (hfCFB) overexpressing ADAMTSL3 (L3) was used as positive control (hfCFB lysates including cell and extracellular matrix (ECM) proteins). ADAMTSL3 detetction on immunoblots is indicated by arrows. **(a)** The commercially available anti-ADAMTSL3 polyclonal antibody HPA03473 (Merck), immunised to aa 1016-1110, sequence ALREPMREYPGMDHSEANSLGVTWHKMRQMWNNKNDLYLDDDHISNQPFLRALLGHCSNSAGSTNS WELKNKQFEAAVKQGAYSMDTAQFDELIR, interspersed between the Ig-like C2-type 1 and Ig-like C2-type 2 domain. **(b)** A custom made antibody for the N-terminal region of ADAMTSL3. Both antibodies detected the positive control, with oo signal in negative control. None of the antibodies detected ADAMTSL3 in mouse LV, while the custom-made antibody was superior to the commercially available in detection of ADAMTSL3 in human LV. Thus, the latter was used in our study in lysates from human LV biopsies and hfCFBs.

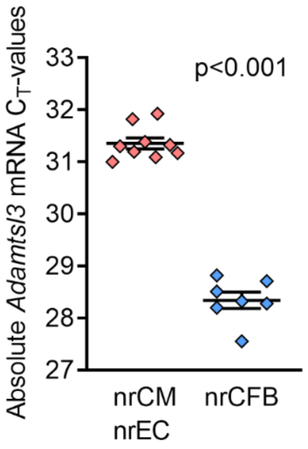

Fig. S2 ***Adamtsl3*** **expression is negligible in primary cardiomyocytes**
Absolute C_T-values for *Adamtsl3* in mRNA samples from primary cultures from neonatal rats (1-3 day old). Cardiac fibroblasts (nrCFB) were separated from cardiomyocytes (nrCM), and endothelial cells (nrEC) were detected in the nrCM fraction[2]. n=3 isolations of n=60 hearts, with n=1-3 culture replicates per isolation. C_T values for *Adamtsl3* in nrCM/nrEC were 31-32, with 20ng input cDNA, indicating low expression. Data are shown as individual value scatter plot with mean ± SEM. Statistical analysis was performed on nrCFBs vs. nrCMs using the Student's *t*-test.

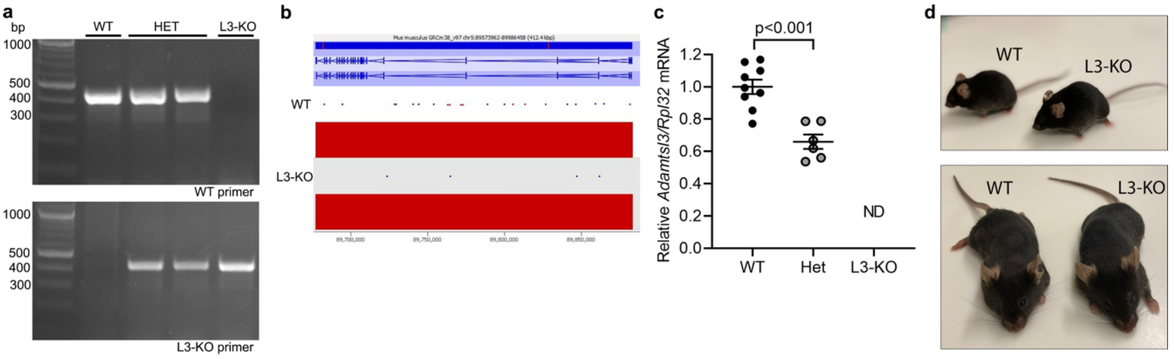

Fig. S3 Characterization of a novel ***Adamtsl3*** **knock-out allele**
Adamtsl3 was disrupted using CRISPR-Cas9 in C57BL/6J mice. Mice hemizygous for the targeted allele were intercrossed to homozygousity to generate *Adamtsl3* knock-out (L3-KO) mice. **(a)** PCR products from genotyping of ear biopsies from wild-type (WT), heterozygous (Het), and L3-KO mice. Genotyping was performed with two PCR reactions using separate WT and L3-KO forward primers and a common reverse primer, that each result in an allele-specific 428 bp PCR product. Genotyping of Hets provided both products, whereas specific PCR products were seen in WTs and L3-KOs as appropriate. **(b)** RNA-seq of WT and L3-KO left ventricular (LV) tissue, with several *Adamtsl3* reads in WT, and negligible numbers in L3-KO. **(c)** RT-qPCR of *Adamtsl3/Rpl32* mRNA in LVs from WT, Het, and L3-KO littermate male mice at baseline (n=9 WT, n=6 Het, n=9 L3-KO). Data are shown as individual value scatterplot with mean ± SEM. Statistical difference between Het vs. WT was tested using the Student's *t*-test. ND, not detected. (d) Images of representative adult WT and L3-KO littermate mice, showing a normal appearance (photos: KB Rypdal).

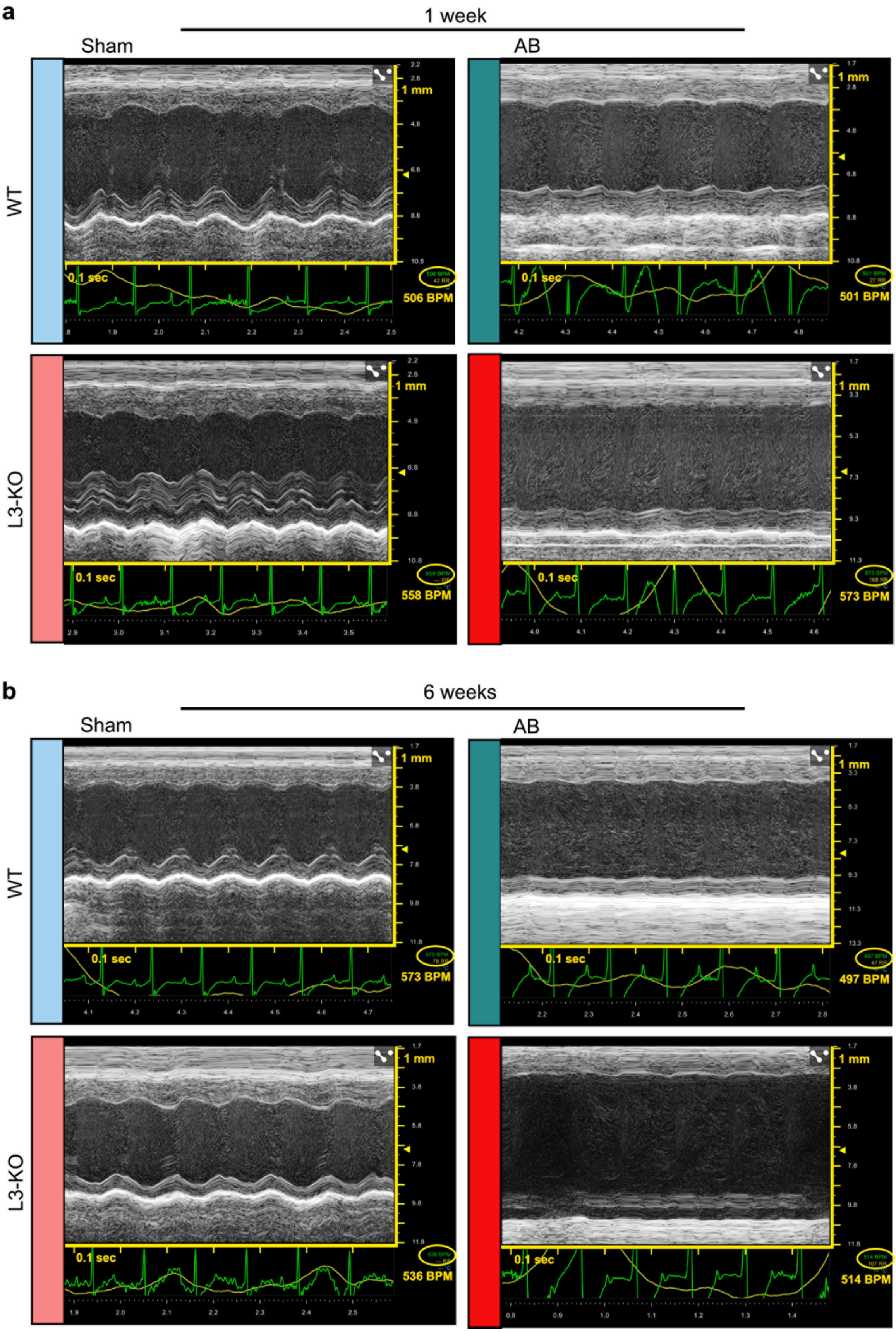

Fig. S4 Representative echocardiography images of left ventricles corresponding to manuscript Fig. 2
M-mode echocardiography images of wild-type (WT) and *Adamtsl3* knock-out (L3-KO) left ventricles **(a)** one and **(b)** six weeks after aortic banding (AB) or sham surgery. Scale: 0.1 seconds, x-axis, 1 mm, y-axis. Electrocardiograms are shown in green beneath the x-axis, and heart rate (BPM) is highlighted in yellow in the lower right corners.

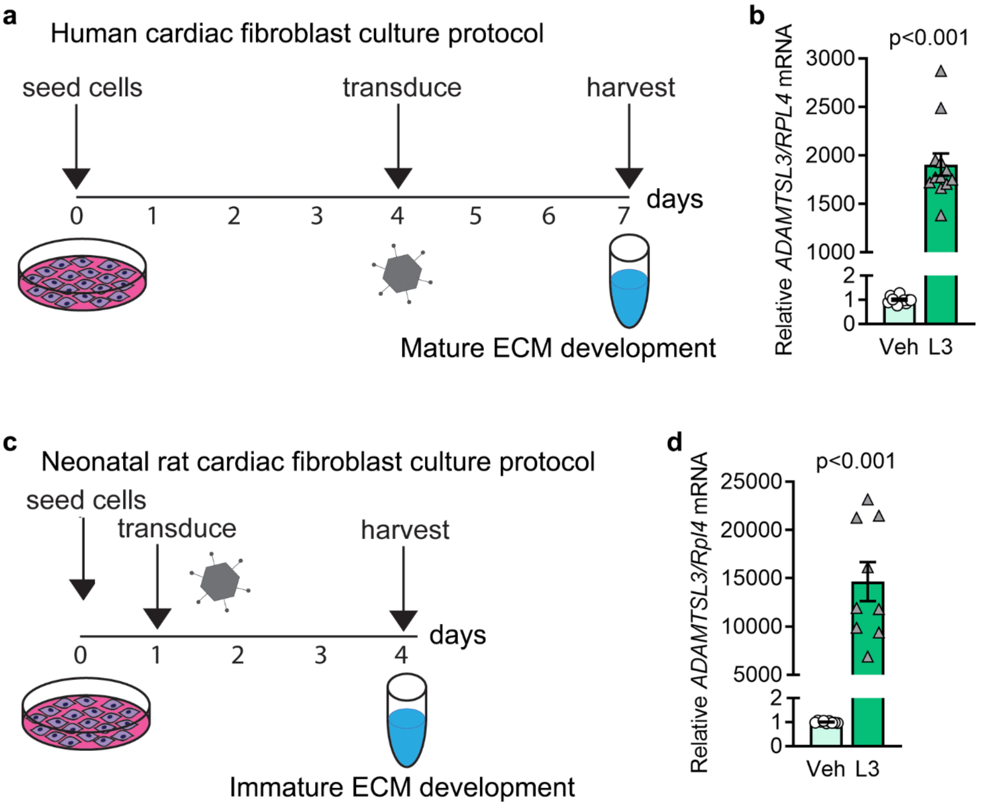

Fig. S5 Experimental strategy and validation of ADAMTSL3 overexpression in neonatal rat primary cardiac fibroblasts and human foetal cardiac fibroblasts

(a) Culture protocol for human foetal CFBs. Cells were cultured for a total of seven days, producing a rich ECM network, with a TGFβ-binding and activating ECM network, as previously described[2]. Cells were transduced with ADAMTSL3 (L3) or control (vehicle, Veh) adenoviruses on day four. **(b)** qPCR of *ADAMTSL3/RPL4* mRNA in hfCFBs transduced with L3 and Veh. Data represent experiments from three different cell passages. **(c)** Culture protocol for primary cardiac fibroblasts (CFBs) isolated from 1-3 days old rat hearts. Cells were cultured for a total of four days, producing a developing, immature extracellular matrix (ECM) network, as previously described[2]. Cells were transduced with L3 or Veh 24 h after seeding. **(d)** qPCR of *ADAMTSL3/Rpl4* mRNA in primary rat CFBs transduced with L3 or Veh (n=3 isolations of n=60 hearts, with n=3 technical replicates per isolation). Data are presented as individual value scatterplots with mean ± SEM. Statistical differences were tested between L3 and Veh using the Student's *t*-test.

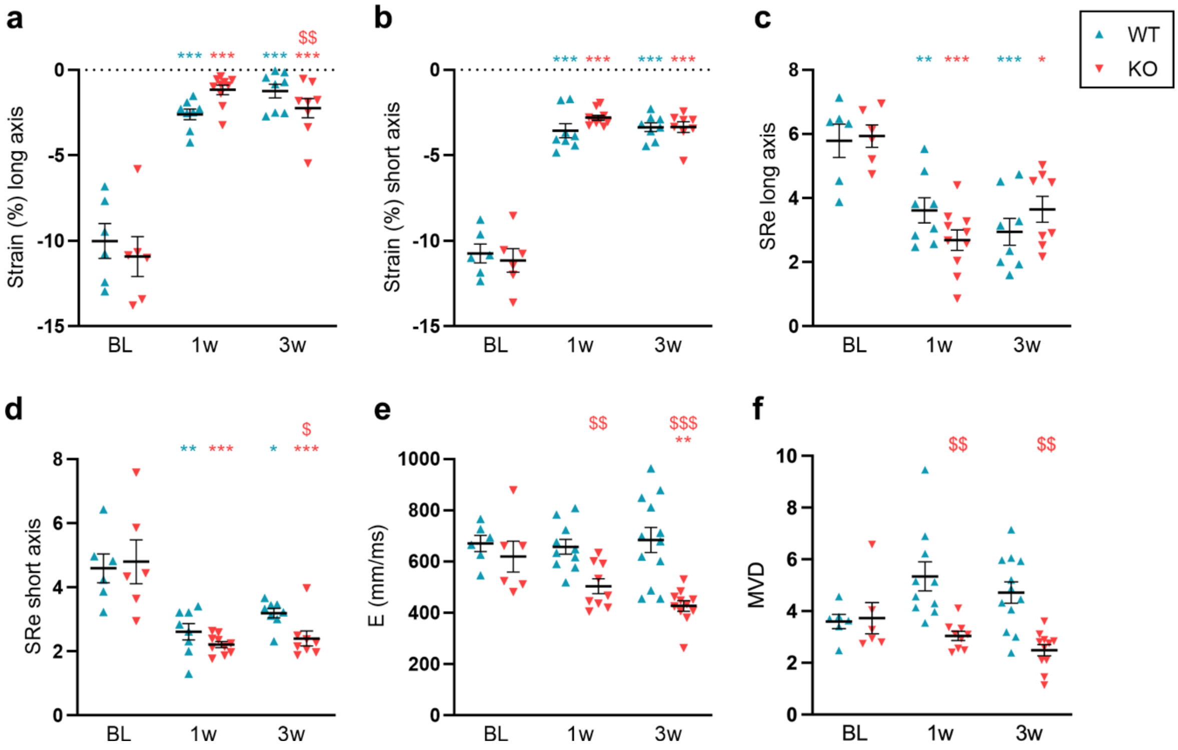

Fig. S6 *Adamtsl3* knock-out mice develop increased tissue strain after aortic banding
(a-b) Mean tissue strain (%) in myocardial long axis (a) and short axis (b) from MRI tissue phase mapping (TPM) recordings, and **(c-d)** peak strain rate (SRe) in long axis (c) and short axis (d) from TPM recordings, measured at baseline (BL), 1 and 3 weeks post aortic banding (AB), in *Adamtsl3* knock-out (KO) and wild-type (WT) mice. **(e)** Peak mitral valve filling velocity in early diastole, and **(f)** mitral valve deceleration (MVD), measured with Doppler echocardiography at BL, 1 and 3 weeks post-AB in KO and WT mice. Data are presented as individual value scatterplots with mean ± SEM. Statistical differences were tested using ordinary one-way ANOVA with Tukey's multiple comparisons test for all groups vs. WT BL at all time-points (*), and the Student's *t*-test for L3-KO vs WT at discrete time-points ($). $^{*/\$}$p<0.05, $^{**/\$\$}$p<0.01, $^{***/\$\$\$}$p<0.001.

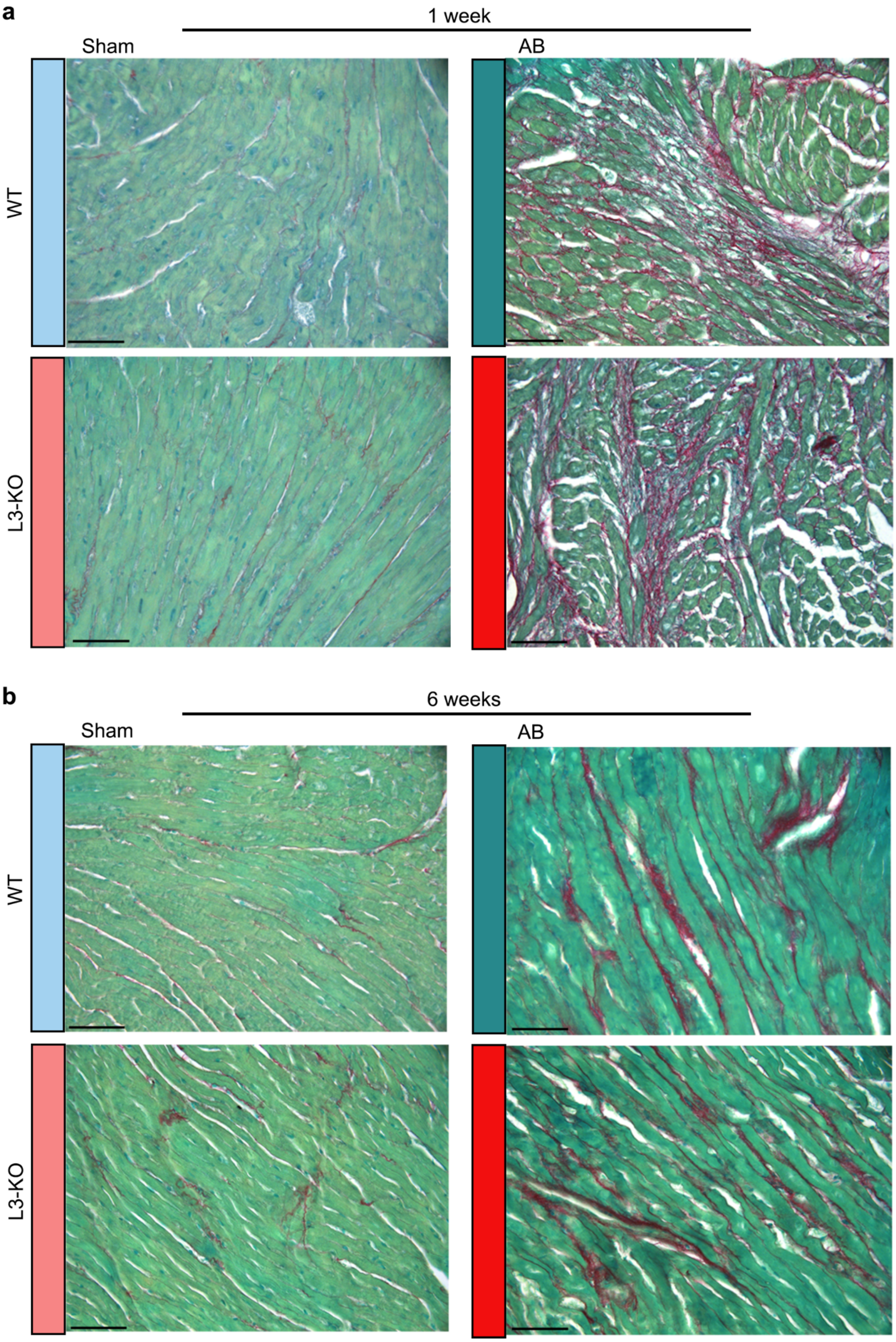

Fig. S7 Representative histology of mid-ventricular sections corresponding to manuscript Fig. 5
Wild-type (WT) and *Adamtsl3* knock-out (L3-KO) mice were subjected to aortic banding (AB) or sham surgery for 1 or 6 weeks. Picrosirius Red, Fast Green, and Alcian Blue (RGB)-staining of mid-ventricular sections of WT and L3-KO left ventricles at 1 **(a)** and 6 **(b)** weeks post-AB, showing collagen in red and cardiac muscle tissue in green. Scale bar = 50µm.

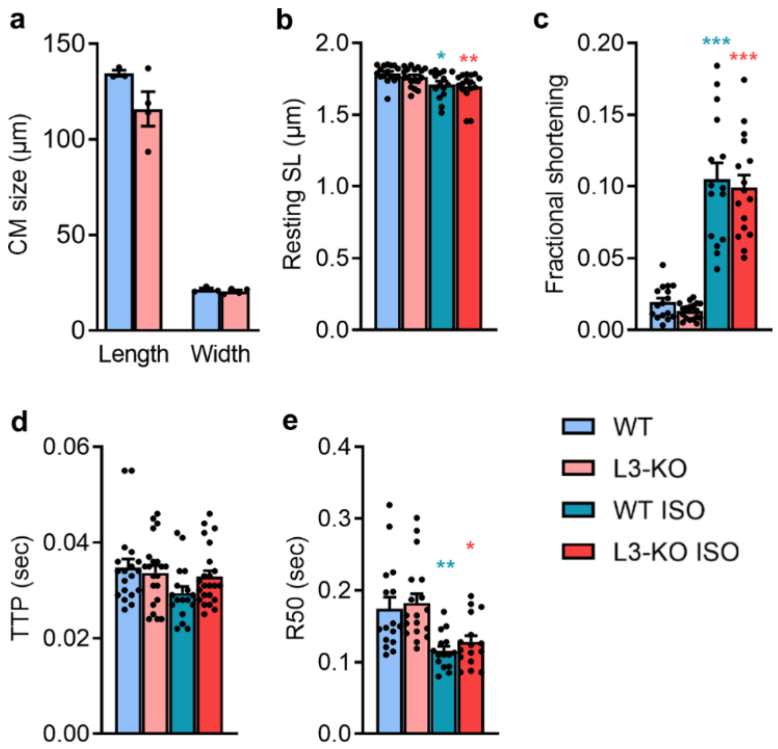

Fig. S8 Wild-type and *Adamtsl3* knock-out cardiomyocytes are similar in size at baseline, with similar contraction at baseline and stress
Isolated cardiomyocytes (CMs) from wild-type (WT, n=3) and *Adamtsl3* knock-out (L3-KO, n=4) mice. **(a)** CM length and width in WT and L3-KO (n=30 cells per animal). **(b)** Resting sarcomere length (SL) in WT and L3-KO at 1 Hz pacing, at baseline (n=16 WT and n=18 L3-KO cells), and after isoproterenol (ISO, n=15 WT and n=16 L3-KO cells). **(c)** CM contraction at 1 Hz pacing at baseline (n=16 WT and n=18 L3-KO cells), and cell shortening after ISO (n=15 WT and n=16 L3-KO cells). **(d-e)** Contraction and relaxation kinetics with **(d)** time-to-peak contraction (TTP) and **(e)** time from peak to 50% relaxation (R50). Data are individual values with mean ± SEM. Statistical analysis was performed using the one-way ANOVA with Dunnett's multiple comparisons test. *p<0.05, **p<0.01, and ***p<0.001 for all groups vs. WT.

Supplementary tables

Table SI Baseline characteristics of untreated *Adamtsl3*-KO and WT mice

	WT	L3-KO
**n (biometry)**	41	45
**BW (g)**	25.39 ±0.45	26.09 ±0.41
**HW (mg)**	129.7 ±2.95	146.4 ±4.17* (13%)
**HW/BW**	5.19 ±0.08	5.67 ±0.12* (9%)
**Tibia length (mm)**	17.04 ±0.05	17.41 ±0.05* (2%)
**n (biometry)**	20	22
**LW (mg)**	151.5 ±4.49	157.3 ±3.37
**LW/BW**	5.79 ±0.18	5.89 ±0.56
**n (echo)**	15	17
**HR (bpm)**	458 ±11.0	458 ±9.74
**LVPWd (mm)**	0.62 ±0.01	0.63 ±0.01
**IVSd (mm)**	0.63 ±0.01	0.62 ±0.01
**LVIDd (mm)**	4.06 ±0.08	4.21 ±0.10
**LVIDs (mm)**	3.13 ±0.09	3.32 ±0.11
**LAD (mm)**	1.30 ±0.03	1.28 ±0.03
**FS (%)**	23.1 ±1.01	21.2 ±1.21
**n (MRI)**	6	5
**LVM (mg)**	84.69 ±3.16	99.68 ±10.68
**EDV (µL)**	47.87 ±3.18	57.93 ±8.17
**EF (%)**	67.80 ±2.34	55.18 ±6.29

Baseline biometrics and cardiac phenotype of untreated C57BL/6 wild-type (WT) and *Adamtsl3* knock-out (L3-KO) mice 8-12 weeks of age. Cardiac structure and function were measured on anesthetized animals breathing 1-2% isoflurane using echocardiography (echo) and cardiac magnetic resonance imaging (MRI). Abbreviations: n, number of animals; BW, body weight; HW, heart weight; HR, heart rate; LVPWd, left ventricular posterior wall thickness in diastole; IVSd, interventricular septum in diastole; LVIDd, left ventricle internal diameter in diastole; LAD, left atrial diameter; FS, fractional shortening; LVM, LV mass; EDV, end diastolic volume; EF, ejection fraction. Data are presented as mean ± SEM. Statistical differences were calculated using the Student's *t*-test, *p<0.05 for L3-KO vs. WT.

Table SII Baseline characteristics of *Adamtsl3*-KO, heterozygote and WT littermate mice

	WT	Het	L3-KO
**n**	11	12	13
**BW (g)**	27.22 ±0.62	27.34 ±0.61	27.97 ±0.71
**HW (mg)**	133.6 ±3.38	134.2 ±4.50	156.2 ±7.97 *(17%) $^{\$}$(16%)
**HW/BW**	4.92 ±0.12	4.90 ±0.13	5.56 ±0.68 *(13%) $^{\$}$(13%)
**Tibia length (mm)**	17.09 ±0.14	17.43 ±0.09	17.56 ±0.10 *(3%)

Biometric parameters of wild-type (WT), heterozygotes (Het) and *Adamtsl3* knock-out (L3-KO) 12-16 weeks old C57BL/6 mice at baseline. % increase of L3-KO values are shown in parentheses. Abbreviations: n, number of animals; BW, body weight; HW, heart weight. Data are presented as mean ± SEM. Statistical differences were calculated using the ordinary ANOVA with Tukey's multiple comparisons test, *p<0.05 L3-KO vs. WT, $^{\$}$p<0.05 L3-KO vs. Het.

Table SIII TaqMan gene expression assays used for qPCR

Gene	Assay name	Species	Gene	Assay name	Species
ACTA2	Hs00426835_g1	Human	***Adamtsl3***	Mm01312414_m1	Mouse
ADAMTSL3	Hs00324954_m1	Human	***Acta2***	Mm00725412_s1	Mouse
COL1A2	Hs01028956_m1	Human	***Col1a1***	Mm00801666_g1	Mouse
COL1A1	Hs00164004_m1	Human	***Col1a2***	Mm00483888_m1	Mouse
COL3A1	Hs00943809_m1	Human	***Col3a1***	Mm00802305_g1	Mouse
CTGF	Hs00170014_m1	Human	***Ctgf***	Mm01192933_g1	Mouse
ELN	Hs00355783_m1	Human	***Fbn1***	Mm00514908_m1	Mouse
FBN1	Hs00171191_m1	Human	***Fbn2***	Mm00515713_m1	Mouse
FBN2	Hs00266592_m1	Human	***Fn1***	Mm01256744_m1	Mouse
FN1	Hs01549976_m1	Human	***Lox***	Mm00495386_m1	Mouse
KI67	Hs00606991_m1	Human	***Ltbp1***	Mm00498234_m1	Mouse
LOX	Hs00942482_g1	Human	***Myh6***	Mm00440359_m1	Mouse
LTBP1	Hs01558763_m1	Human	***Myh7***	Mm00600555_m1	Mouse
MCM2	Hs01091564_m1	Human	***Nppa***	Mm01255747_g1	Mouse
PCNA	Hs00427214_g1	Human	***Nppb***	Mm01255770_g1	Mouse
POSTN	Hs01566750_m1	Human	***Postn***	Mm01284913_g1	Mouse
RPL32	Hs00851655_g1	Human	***Rpl32***	Mm02528467_g1	Mouse
RPL4	Hs03044646_g1	Human	***Spp1***	Mm00436767_m1	Mouse
SPP1	Hs00959010_m1	Human	***Tgfb1***	Mm01178820_m1	Mouse
TGFB1	Hs00998133_m1	Human	***Adamtsl3***	Rn01477616_m1	Rat

Supplementary references

1 Hall, N. G., Klenotic, P., Anand-Apte, B. & Apte, S. S. ADAMTSL-3/punctin-2, a novel glycoprotein in extracellular matrix related to the ADAMTS family of metalloproteases. *Matrix Biol.* **22**, 501-510 (2003).

2 Rypdal, K. B. *et al.* The extracellular matrix glycoprotein ADAMTSL2 is increased in heart failure and inhibits TGFbeta signalling in cardiac fibroblasts. *Sci. Rep.* **11**, 19757 (2021). https://doi.org:10.1038/s41598-021-99032-2

Supplementary blots

Fig. 1 blots

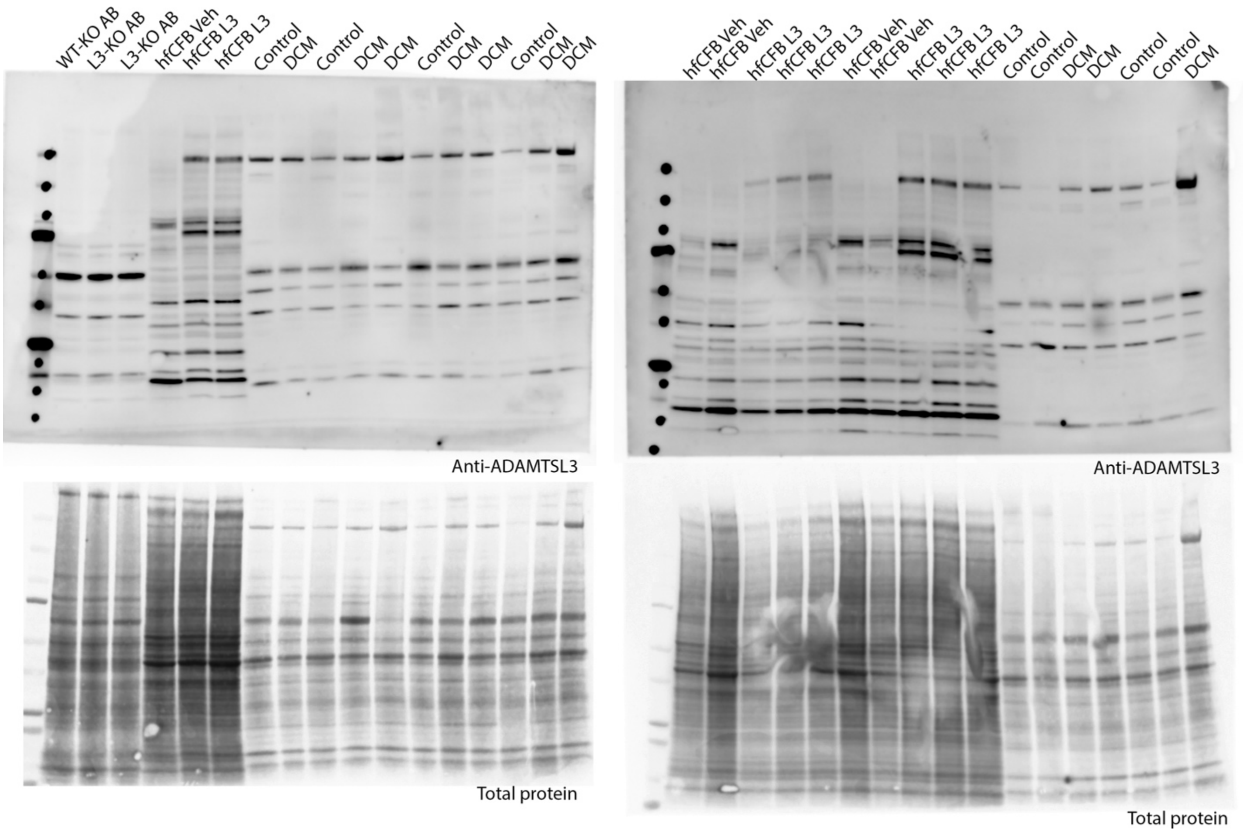

Fig. 4 blots

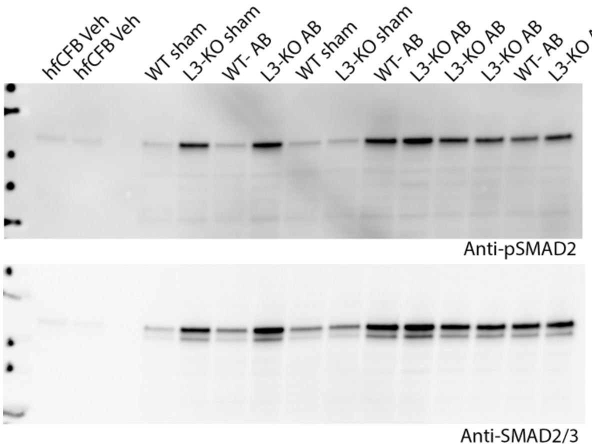
hfCFB Veh
hfCFB Veh
WT sham
L3-KO sham
WT- AB
L3-KO AB
WT sham
L3-KO sham
WT- AB
L3-KO AB
L3-KO AB
L3-KO AB
WT- AB
L3-KO AB
Anti-pSMAD2
Anti-SMAD2/3

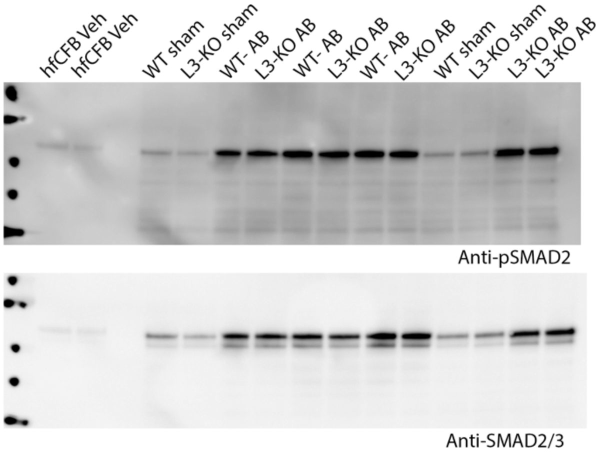
hfCFB Veh
hfCFB Veh
WT sham
L3-KO sham
WT- AB
L3-KO AB
WT- AB
L3-KO AB
WT- AB
L3-KO AB
WT sham
L3-KO sham
L3-KO AB
L3-KO AB
Anti-pSMAD2
Anti-SMAD2/3

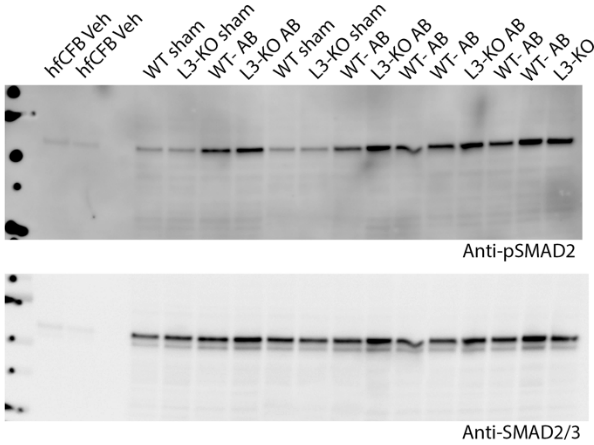
hfCFB Veh
hfCFB Veh
WT sham
L3-KO sham
WT- AB
L3-KO AB
WT sham
L3-KO sham
WT- AB
L3-KO AB
WT- AB
WT- AB
L3-KO AB
WT- AB
WT- AB
L3-KO AB
Anti-pSMAD2
Anti-SMAD2/3

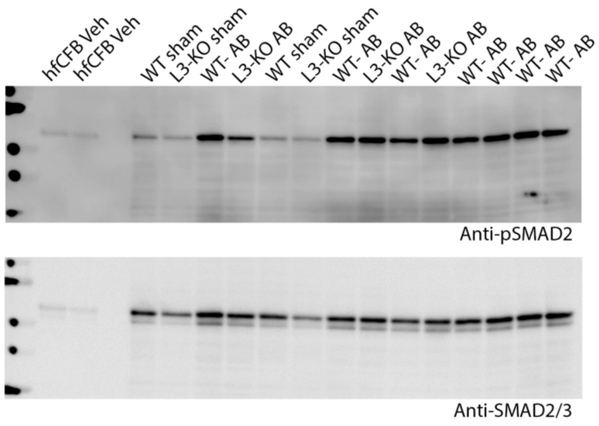
hfCFB Veh
hfCFB Veh
WT sham
L3-KO sham
WT- AB
L3-KO AB
WT sham
L3-KO sham
WT- AB
L3-KO AB
WT- AB
L3-KO AB
WT- AB
WT- AB
WT- AB
WT- AB
Anti-pSMAD2
Anti-SMAD2/3

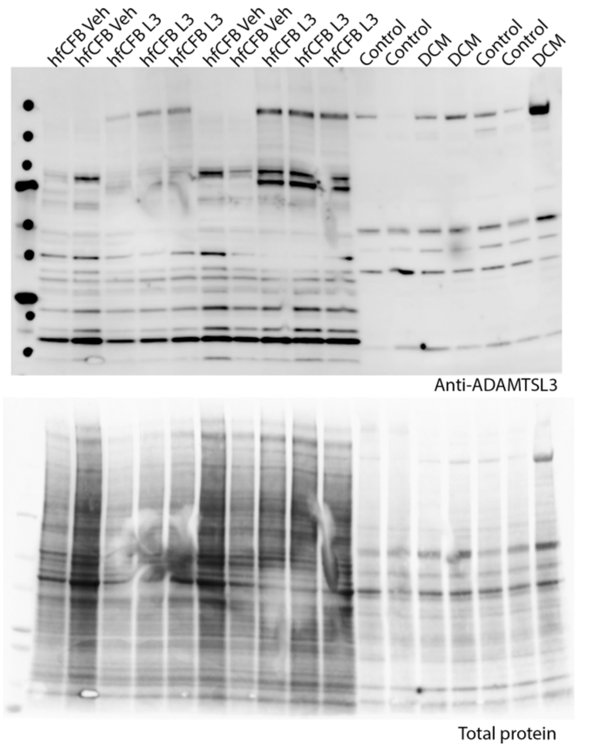
hfCFB Veh
hfCFB Veh
hfCFB L3
hfCFB L3
hfCFB L3
hfCFB Veh
hfCFB Veh
hfCFB L3
hfCFB L3
hfCFB L3
Control
Control
DCM
DCM
Control
Control
DCM
Anti-ADAMTSL3
Total protein

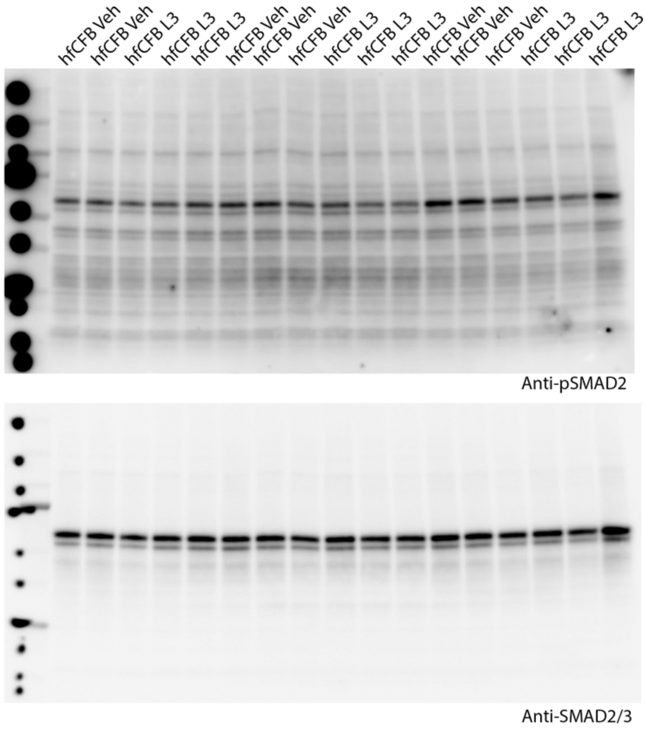
hfCFB Veh
hfCFB Veh
hfCFB L3
hfCFB L3
hfCFB L3
hfCFB Veh
hfCFB Veh
hfCFB Veh
hfCFB L3
hfCFB L3
hfCFB L3
hfCFB Veh
hfCFB Veh
hfCFB Veh
hfCFB L3
hfCFB L3
hfCFB L3
Anti-pSMAD2
Anti-SMAD2/3

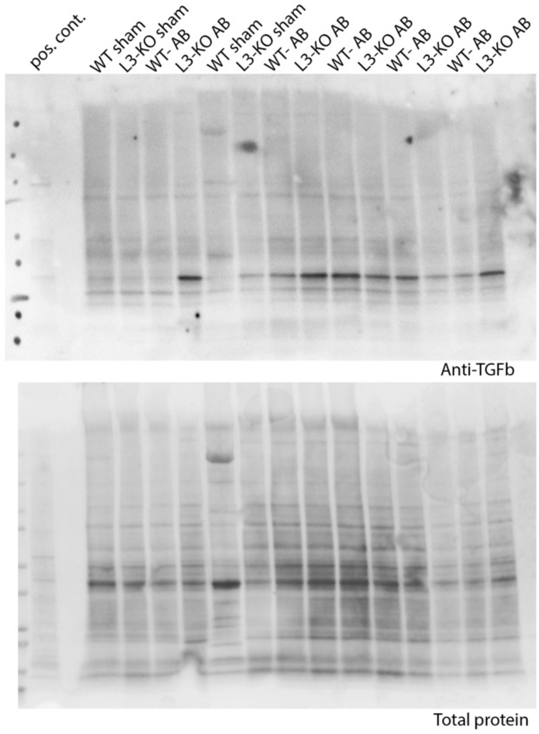
pos. cont.
WT sham
L3-KO sham
WT- AB
L3-KO AB
WT sham
L3-KO sham
WT- AB
L3-KO AB
WT- AB
L3-KO AB
WT- AB
L3-KO AB
WT- AB
L3-KO AB
Anti-TGFb
Total protein

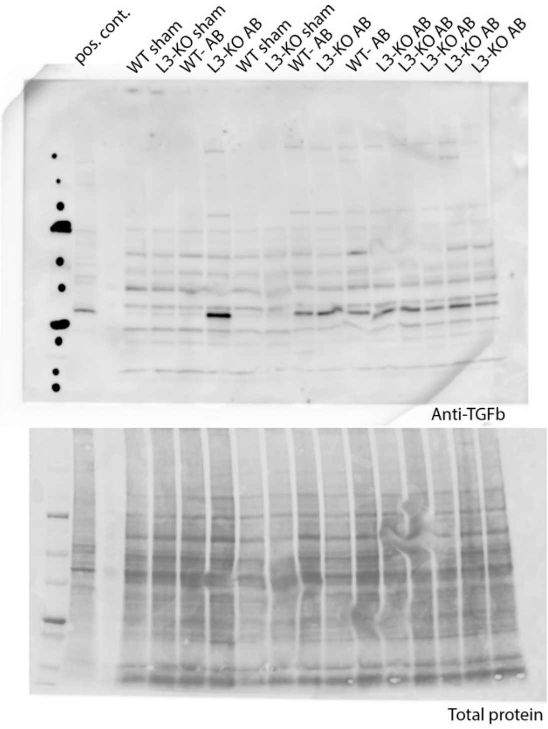
pos. cont.
WT sham
L3-KO sham
WT- AB
L3-KO AB
WT sham
L3-KO sham
WT- AB
L3-KO AB
WT- AB
L3-KO AB
L3-KO AB
L3-KO AB
L3-KO AB
L3-KO AB
Anti-TGFb
Total protein

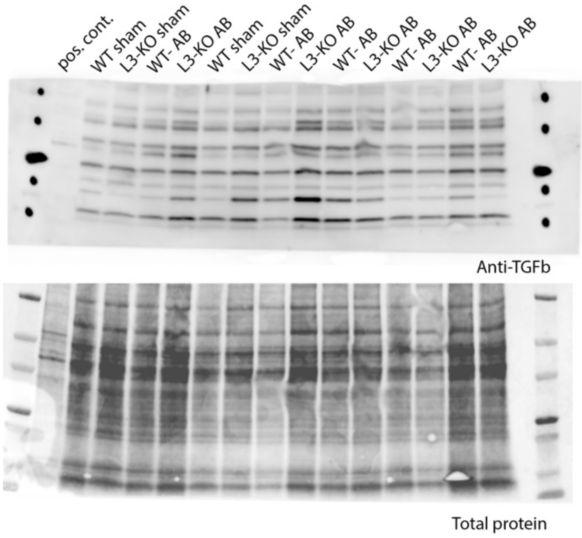
pos. cont.
WT sham
L3-KO sham
WT- AB
L3-KO AB
WT sham
L3-KO sham
WT- AB
L3-KO AB
WT- AB
L3-KO AB
WT- AB
L3-KO AB
WT- AB
L3-KO AB
Anti-TGFb
Total protein

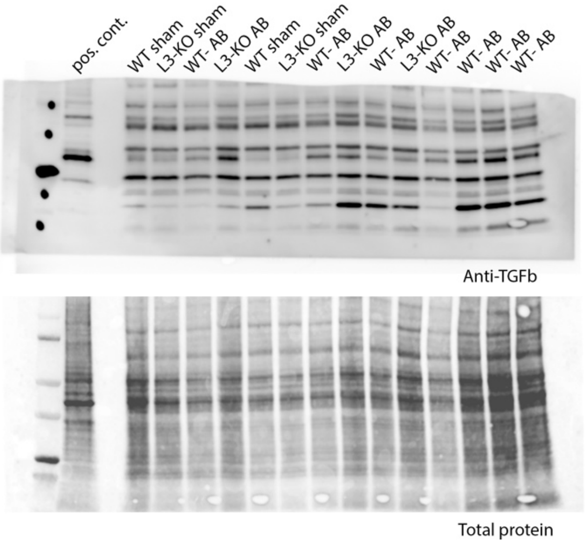
pos. cont.
WT sham
L3-KO sham
WT- AB
L3-KO AB
WT sham
L3-KO sham
WT- AB
L3-KO AB
WT- AB
L3-KO AB
WT- AB
WT- AB
WT- AB
WT- AB
Anti-TGFb
Total protein

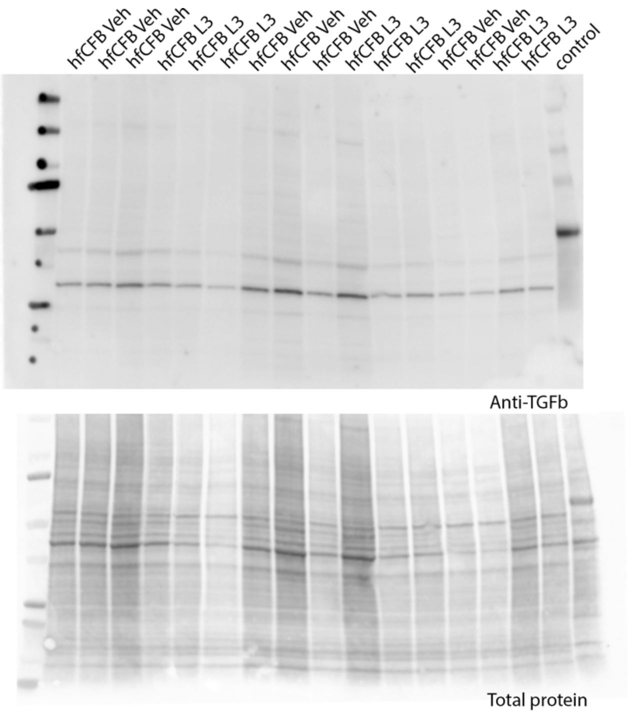
Anti-TGFb
Total protein

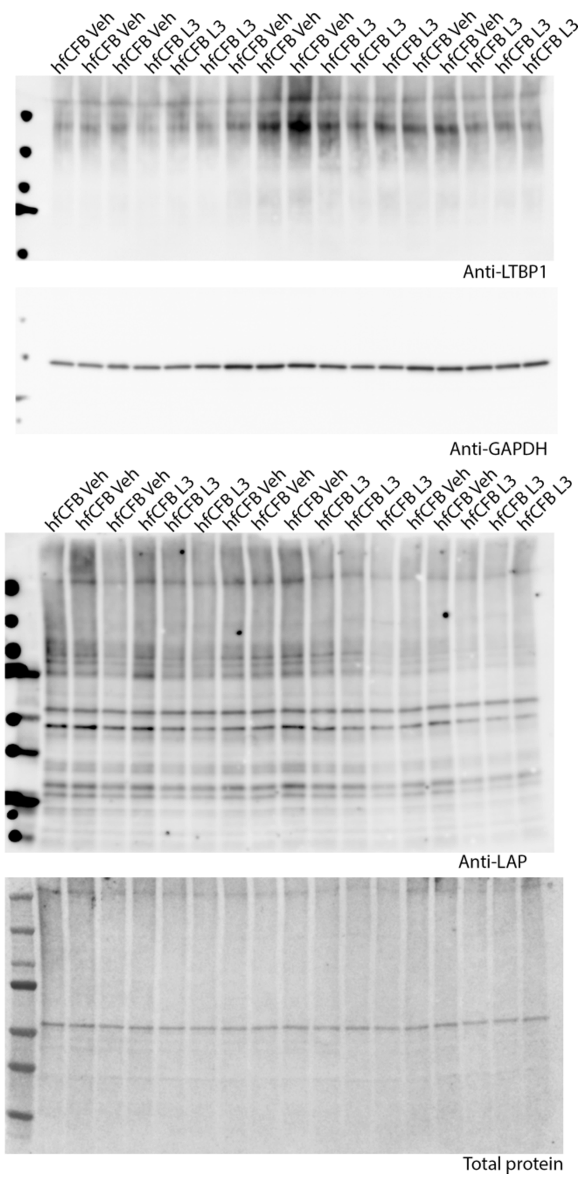
Anti-LTBP1
Anti-GAPDH
Anti-LAP
Total protein

Fig. 6 blots

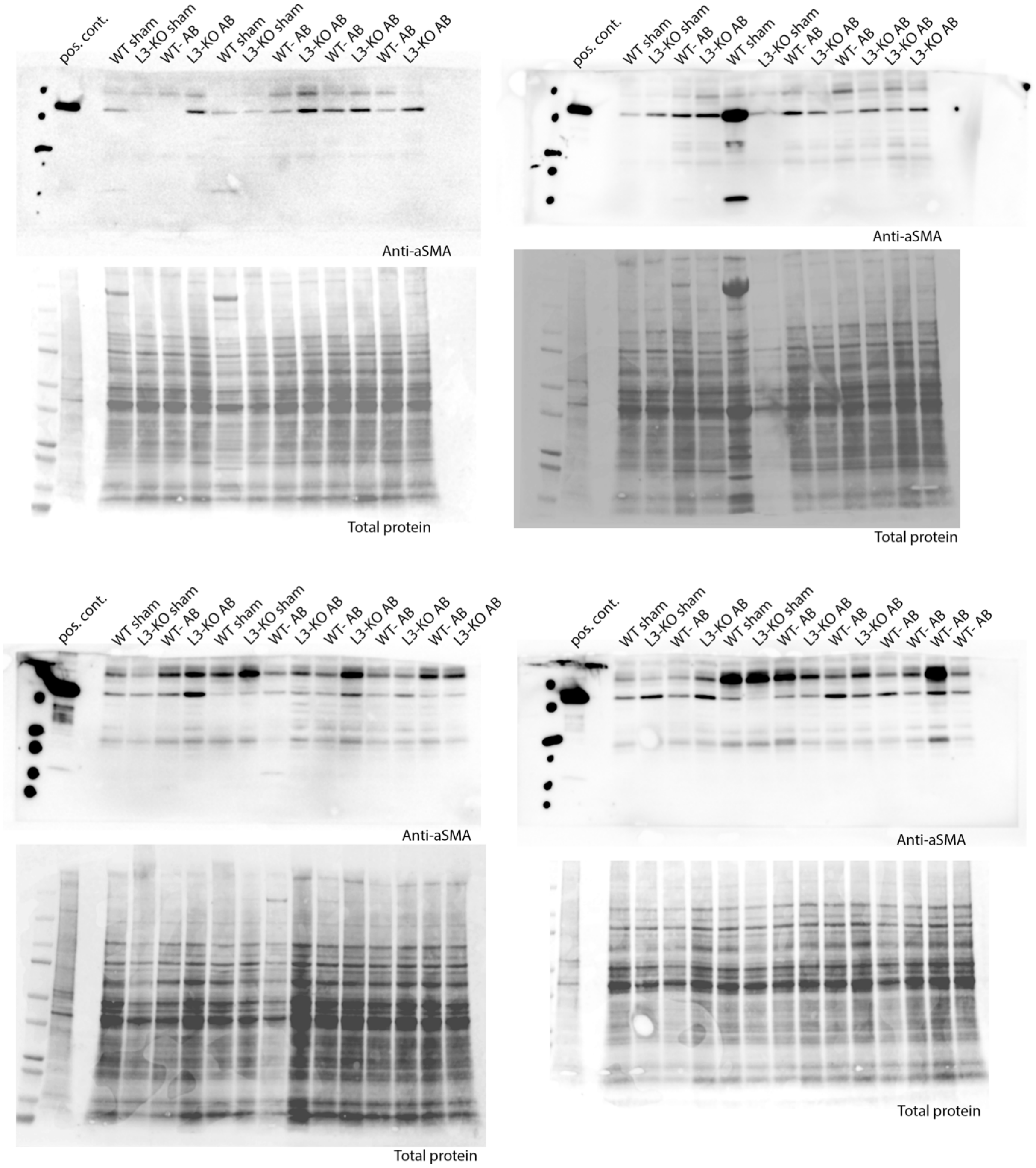
pos. cont.
WT sham
L3-KO sham
WT- AB
L3-KO AB
WT sham
L3-KO sham
WT- AB
L3-KO AB
WT- AB
L3-KO AB
WT- AB
L3-KO AB
Anti-aSMA
Total protein
pos. cont.
WT sham
L3-KO sham
WT- AB
L3-KO AB
WT sham
L3-KO sham
WT- AB
L3-KO AB
WT- AB
L3-KO AB
L3-KO AB
L3-KO AB
Anti-aSMA
Total protein
pos. cont.
WT sham
L3-KO sham
WT- AB
L3-KO AB
WT sham
L3-KO sham
WT- AB
L3-KO AB
WT- AB
L3-KO AB
WT- AB
L3-KO AB
WT- AB
L3-KO AB
Anti-aSMA
Total protein
pos. cont.
WT sham
L3-KO sham
WT- AB
L3-KO AB
WT sham
L3-KO sham
WT- AB
L3-KO AB
WT- AB
L3-KO AB
WT- AB
WT- AB
WT- AB
WT- AB
Anti-aSMA
Total protein

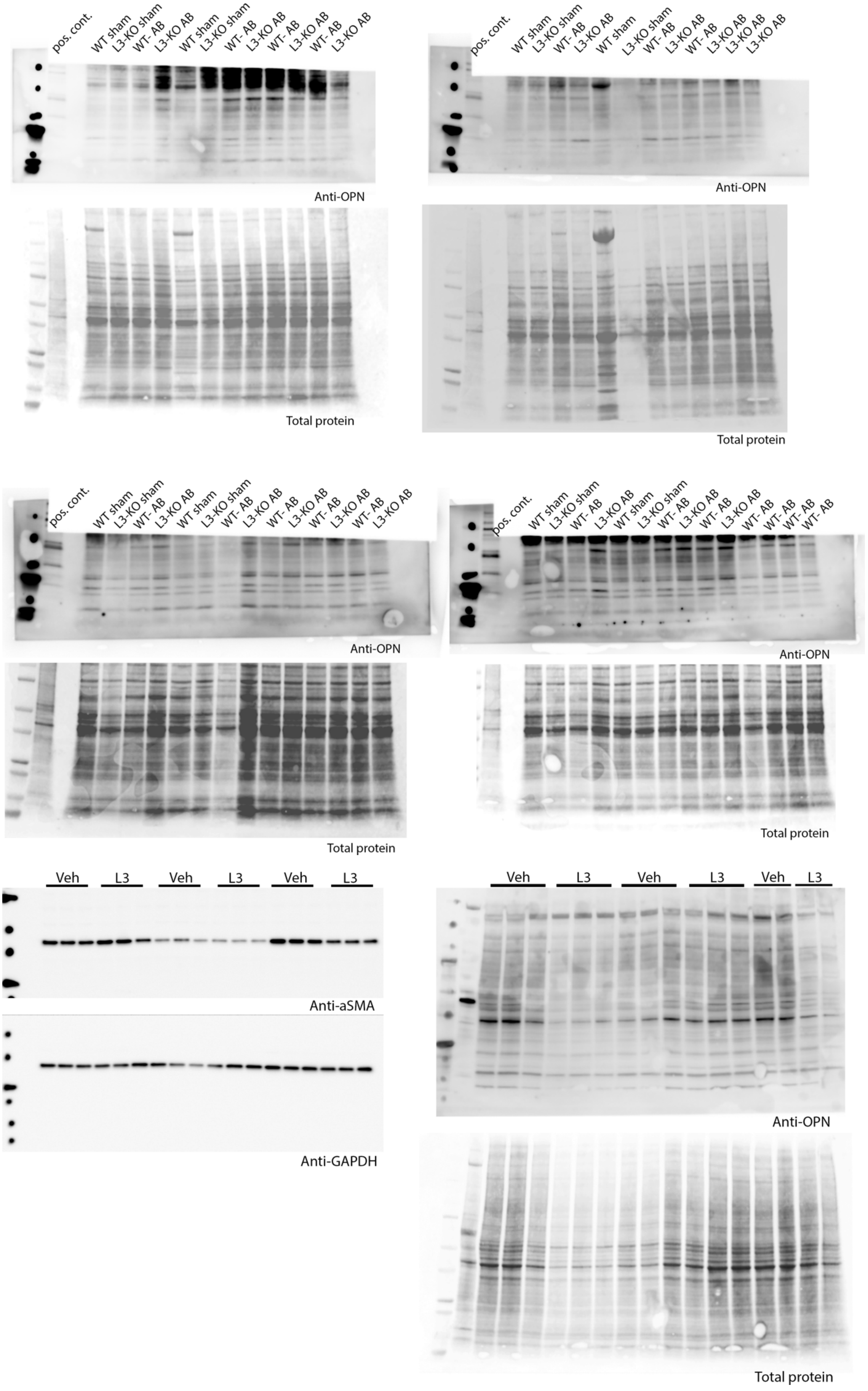
pos. cont.
WT sham
L3-KO sham
WT- AB
L3-KO AB
WT sham
L3-KO sham
WT- AB
L3-KO AB
WT- AB
L3-KO AB
WT- AB
L3-KO AB
Anti-OPN
Total protein
pos. cont.
WT sham
L3-KO sham
WT- AB
L3-KO AB
WT sham
L3-KO sham
WT- AB
L3-KO AB
WT- AB
L3-KO AB
L3-KO AB
L3-KO AB
Anti-OPN
Total protein
pos. cont.
WT sham
L3-KO sham
WT- AB
L3-KO AB
WT sham
L3-KO sham
WT- AB
L3-KO AB
WT- AB
L3-KO AB
WT- AB
L3-KO AB
WT- AB
L3-KO AB
Anti-OPN
Total protein
pos. cont.
WT sham
L3-KO sham
WT- AB
L3-KO AB
WT sham
L3-KO sham
WT- AB
L3-KO AB
WT- AB
L3-KO AB
WT- AB
WT- AB
WT- AB
WT- AB
Anti-OPN
Total protein
Veh
L3
Veh
L3
Veh
L3
Anti-aSMA
Anti-GAPDH
Veh
L3
Veh
L3
Veh
L3
Anti-OPN
Total protein
