# Supplementary material for: ADAMTSL3 knock-out mice develop cardiac dysfunction and dilatation with increased TGFβ signalling after pressure overload
Source: Commun Biol. 2022 Dec 20;5:1392. doi: 10.1038/s42003-022-04361-1 (PMC9767913; doi:10.1038/s42003-022-04361-1)
Supplement: Supplementary file 2 — Description of Additional Supplementary Data [file 42003_2022_4361_MOESM2_ESM.docx]

**Description of Additional Supplementary Files**

**File name:** Supplementary Data 1

**Description:** A Supplementary Data file with RNA sequencing data (Figure 3) on LV tissue from WT and L3-KO mice at one week post-AB, containing the following sheets:

· Annotated DEGs with FDR<0.05 · Enriched KEGG pathways from DAVID

· Enriched GO biological processes from DAVID

· Enriched GO molecular functions from DAVID

· Enriched GO cellular components from DAVID

· Ingenuity Pathway Analysis (IPA) from QIAGEN

**File name:** Supplementary Data 2

**Description:** The source data behind the graphs in the main figures (Figure 1, 2, 4, 5, 6)
