# Supplementary material for: ADAMTSL3 knock-out mice develop cardiac dysfunction and dilatation with increased TGFβ signalling after pressure overload
Source: Commun Biol. 2022 Dec 20;5:1392. doi: 10.1038/s42003-022-04361-1 (PMC9767913; doi:10.1038/s42003-022-04361-1)
Supplement: Supplementary file 5 — Nature reporting summary [file 42003_2022_4361_MOESM5_ESM.pdf]

nature portfolio

Corresponding author(s): Karoline Bjarnesdatter Rypdal

Last updated by author(s): 30.11.22

Reporting Summary

Nature Portfolio wishes to improve the reproducibility of the work that we publish. This form provides structure for consistency and transparency in reporting. For further information on Nature Portfolio policies, see our Editorial Policies and the Editorial Policy Checklist.

Statistics

For all statistical analyses, confirm that the following items are present in the figure legend, table legend, main text, or Methods section.

n/a	Confirmed	
☐	☒	The exact sample size (*n*) for each experimental group/condition, given as a discrete number and unit of measurement
☐	☒	A statement on whether measurements were taken from distinct samples or whether the same sample was measured repeatedly
☐	☒	The statistical test(s) used AND whether they are one- or two-sided<br>*Only common tests should be described solely by name; describe more complex techniques in the Methods section.*
☒	☐	A description of all covariates tested
☐	☒	A description of any assumptions or corrections, such as tests of normality and adjustment for multiple comparisons
☐	☒	A full description of the statistical parameters including central tendency (e.g. means) or other basic estimates (e.g. regression coefficient) AND variation (e.g. standard deviation) or associated estimates of uncertainty (e.g. confidence intervals)
☐	☒	For null hypothesis testing, the test statistic (e.g. *F*, *t*, *r*) with confidence intervals, effect sizes, degrees of freedom and *P* value noted<br>*Give P values as exact values whenever suitable.*
☒	☐	For Bayesian analysis, information on the choice of priors and Markov chain Monte Carlo settings
☒	☐	For hierarchical and complex designs, identification of the appropriate level for tests and full reporting of outcomes
☒	☐	Estimates of effect sizes (e.g. Cohen's *d*, Pearson's *r*), indicating how they were calculated

Our web collection on statistics for biologists contains articles on many of the points above.

Software and code

Policy information about availability of computer code

Data collection	No mathematical algorithm or custom code was applied
Data analysis	All data was analyzed using GraphPad Prism v. 9.3.1, additionally, Matlab software was used for MRI data and SeqMonk and the R-based software DESeq2 was used for RNA-seq data.

For manuscripts utilizing custom algorithms or software that are central to the research but not yet described in published literature, software must be made available to editors and reviewers. We strongly encourage code deposition in a community repository (e.g. GitHub). See the Nature Portfolio guidelines for submitting code & software for further information.

Data

Policy information about availability of data

All manuscripts must include a data availability statement. This statement should provide the following information, where applicable:

- Accession codes, unique identifiers, or web links for publicly available datasets
- A description of any restrictions on data availability
- For clinical datasets or third party data, please ensure that the statement adheres to our policy

The RNA-seq data used to generate the plots and charts of Figure 3 are uploaded in Supplementary Data 1, and was deposited in its entirety to the European Nucleotide Archive (ENA, EMBL-EBI, Wellcome Genome Campus, Hinxton, Cambridgeshire, UK), accession: PRJEB47017. All numerical source data used for

generating the main figures are uploaded Supplementary Data 2. Unedited western blot images used to generate figures are presented under Supplementary Blots in the Supplementary Information. All other data are available from the corresponding author on reasonable request.

Human research participants

Policy information about studies involving human research participants and Sex and Gender in Research.

Reporting on sex and gender	Patient and donor characteristics were previously described in Herum et al., 2020, and Melleby et al., 2016, and the patient population includes heart failure patients of both male and female sex. Sex and gender was not considered for the study design, as the samples were collected for other projects, and re-used for this project.
Population characteristics	Patient tissue (human cardiac biopsy samples) was used from biobank tissue from two previously published cohorts; patients with aortic stenosis, LV free wall biopsies were taken during open-heart surgery for aortic valve replacement. Control LV biopsies were taken from normally contracting tissue of patients undergoing surgery for coronary artery disease. Patient characteristics were previously described (Herum et al., 2020). LV biopsies from patients with DCM (n=20) were explanted hearts. LV tissue from non-diseased hearts considered for transplantation, but rejected due to surgical reasons, served as controls. Patient and donor characteristics were described previously (Melleby et al., 2016).
Recruitment	Patients were recruited for previously published studies (Melleby et al., 2016, and Herum et al., 2020), and the samples were re-used for this study.
Ethics oversight	The use of human cardiac biopsies was approved by the Regional Committee for Medical Research Ethics (REK ID 07482a, and 2010/2226) the South-Eastern Regional Health Authority, Norway, and is in accordance with the Declaration of Helsinki. All patients, or the next of kin of donor controls, signed a written informed consent. The biopsies were obtained at Oslo University Hospital (OUH), and all patients received standard clinical evaluation, treatment and follow-up in accordance with hospital guidelines.

Note that full information on the approval of the study protocol must also be provided in the manuscript.

Field-specific reporting

Please select the one below that is the best fit for your research. If you are not sure, read the appropriate sections before making your selection.

☒ Life sciences ☐ Behavioural & social sciences ☐ Ecological, evolutionary & environmental sciences

For a reference copy of the document with all sections, see nature.com/documents/nr-reporting-summary-flat.pdf

Life sciences study design

All studies must disclose on these points even when the disclosure is negative.

Sample size	The sample sizes were determined based on a pilot study, and long-term previous experience.
Data exclusions	Samples were excluded based on pre-defined criteria for exclusion. For animals, HR<400 in echo/MRI analyses qualified for data exclusion due to heavy sedation. Molecular biology samples were excluded based on quality criteria, such as mRNA degradation or similar.
Replication	Animal experiments were performed in three time-distinct cohorts to ensure reproducibility. All cell culture experiments were performed in three or more time-distinct biological replicates with several technical replicates within each round.
Randomization	Animals were stratified based on age and weight, and randomized to aortic banding or sham operation for both KO and WT groups.
Blinding	All animal operations, examinations and data analysis was performed blinded to genotype.

Reporting for specific materials, systems and methods

We require information from authors about some types of materials, experimental systems and methods used in many studies. Here, indicate whether each material, system or method listed is relevant to your study. If you are not sure if a list item applies to your research, read the appropriate section before selecting a response.

March 2021

Materials & experimental systems

n/a	Involved in the study
☐	☒ Antibodies
☐	☒ Eukaryotic cell lines
☒	☐ Palaeontology and archaeology
☐	☒ Animals and other organisms
☒	☐ Clinical data
☒	☐ Dual use research of concern

Methods

n/a	Involved in the study
☒	☐ ChIP-seq
☒	☐ Flow cytometry
☒	☐ MRI-based neuroimaging

Antibodies

Antibodies used	The following antibodies were used: anti-phospho-Smad2 (Ser465/467, Cat # 3108, Cell Signaling), anti-Smad2/3 (Cat # 3102, 8685, Cell Signaling), both diluted 1:1000 in 5% milk, anti-TGFb (Ab92486, Abcam) 1:1000, 1% casein, anti-LAP (AF-246, R&D Systems) 1:1000 in 5% milk under non-reducing conditions, anti-LTBP1 (Cat # MAB-388, R&D Systems) diluted 1:1000 in 5% milk under non-reducing conditions, anti-α-SMA (Cat # A5228, Sigma) diluted 1:10,000 in 5% milk, anti-OPN (Ab181440, Abcam) 1:1000 in 1% milk, anti-GAPDH (Cat # sc-32233, Santa Cruz Biotechnology) diluted 1:1000 in 5% milk, anti-ADAMTSL3 (HPA034773, Atlas Antibodies) 1:1000 in 1% casein, and anti-ADAMTSL3 (in-house polyclonal antibody produced in rabbit) 1:1000 in 1% casein. Secondary antibodies for mouse or rabbit (1:2000, Santa Cruz Biotechnology) was used.
Validation	All antibodies used in this study, except one in-house ADAMTSL3 antibody, are commercially available, with several citations each. They are also extensively used in our lab, with positive and negative controls. The in-house custom made ADAMTSL3 antibody was verified by demonstration in the presented Supplemental Material.

Eukaryotic cell lines

Policy information about cell lines and Sex and Gender in Research

Cell line source(s)	Human foetal cardiac fibroblasts (hfCFBs) were obtained from Cell Applications, Inc (Cat# 306-05f)
Authentication	The cells have not been authenticated after purchase.
Mycoplasma contamination	The cell lines were routinley screened for mycoplasma.
Commonly misidentified lines (See ICLAC register)	*Name any commonly misidentified cell lines used in the study and provide a rationale for their use.*

Animals and other research organisms

Policy information about studies involving animals; ARRIVE guidelines recommended for reporting animal research, and Sex and Gender in Research

Laboratory animals	Mus musculus, C57BL/6, 8-12 week old male Adamtsl3-KO and WT littermates.
Wild animals	The study did not involve wild animals.
Reporting on sex	For this study, only male mice were used for heart failure experiments, to ensure standardized degree of constriction of the aorta.
Field-collected samples	The study did not involve samples collected from the field.
Ethics oversight	The mouse models and experiments were approved by the Norwegian National Animal Research Committee (approval ID 16614) and were in accordance with the NIH Guide for the Care and Use of Laboratory Animals, as well as the ARRIVE guidelines for reporting animal research.

Note that full information on the approval of the study protocol must also be provided in the manuscript.
